# MicroRNAs regulate innate immunity against uropathogenic and commensal-like *Escherichia coli* infections in the surrogate insect model *Galleria mellonella*

**DOI:** 10.1038/s41598-020-59407-3

**Published:** 2020-02-13

**Authors:** Krishnendu Mukherjee, Daniel Amsel, Miriam Kalsy, Andre Billion, Ulrich Dobrindt, Andreas Vilcinskas

**Affiliations:** 1 Fraunhofer Institute for Molecular Biology and Applied Ecology, Department of Bioresources, Winchester Str. 2, 35394 Giessen, Germany; 2 Institute for Insect Biotechnology, Justus Liebig University, Heinrich-Buff-Ring 26-32, 35392 Giessen, Germany; 3 Institute of Hygiene, University of Münster, Mendel Strasse 7, 48149 Münster, Germany

**Keywords:** Epigenomics, Infection, Bacterial immune evasion

## Abstract

Uropathogenic *Escherichia coli* (UPEC) strains cause symptomatic urinary tract infections in humans whereas commensal-like *E. coli* strains in the urinary bladder cause long-term asymptomatic bacteriuria (ABU). We previously reported that UPEC and ABU strains differentially regulate key DNA methylation and histone acetylation components in the surrogate insect host *Galleria mellonella* to epigenetically modulate innate immunity-related gene expression, which in turn controls bacterial growth. In this follow-up study, we infected *G. mellonella* larvae with UPEC strain CFT073 or ABU strain 83972 to identify differences in the expression of microRNAs (miRNAs), a class of non-coding RNAs that regulate gene expression at the post-transcriptional level. Our small RNA sequencing analysis showed that UPEC and ABU infections caused significant changes in the abundance of miRNAs in the larvae, and highlighted the differential expression of 147 conserved miRNAs and 95 novel miRNA candidates. We annotated the *G. mellonella* genome sequence to investigate the miRNA-regulated expression of genes encoding antimicrobial peptides, signaling proteins, and enzymatic regulators of DNA methylation and histone acetylation in infected larvae. Our results indicate that miRNAs play a role in the epigenetic reprograming of innate immunity in *G. mellonella* larvae to distinguish between pathogenic and commensal strains of *E. coli*.

## Introduction

Urinary tract infections (UTIs) are a global public health problem, with 50% of all women experiencing a symptomatic UTI episode at least once in their lifetime. This results in 11 million medical visits and 100,000 hospital admissions in the United States every year^1,2^. Uropathogenic *Escherichia coli* (UPEC) strains cause 70–90% of all UTIs in humans, and antibiotics are the front-line treatment option despite growing resistance among the target strains. UPEC strains infect the urinary bladder through the urethra (cystitis), and if they remain untreated, the infection can spread to the kidneys (pyelonephritis) leading to renal failure and sepsis. Unlike UPEC strains, commensal-like *E. coli* strains can colonize the urinary bladder in large numbers without symptoms. Such asymptomatic bacteriuria (ABU) strains have evolved from UPEC strains by losing the ability to express functional virulence factors^3–6^. The ABU *E. coli* strain 83972 achieves long-term growth in the urinary bladder by adopting a commensal-like lifestyle. It blocks disease-associated signaling pathways and prevents symptomatic UTIs caused by more virulent UPEC strains^7–9^.

Innate immunity-related gene expression distinguishes between infections caused by ABU and UPEC strains in the urinary bladder. Bacterial molecular recognition patterns frequently expressed by bacterial pathogens activate different signaling pathways involved in innate immune response. Toll-like receptor (TLR) 4-mediated signaling distinguishes pathogenic from commensal strains and controls the downstream signaling pathways thus maintaining pathogen specificity^10–12^. Additionally, the secreted TIR domain homologue TcpC is expressed by many UPEC strains and inhibits MyD88 and inflammasome activation^13,14^. ABU strains have also been shown to modulate host gene expression by suppressing RNA polymerase II^7,9^. Surprisingly, the discriminatory host response is not restricted to humans and also occurs in the greater wax moth *Galleria mellonella*, which has been established as a surrogate insect model host to study human pathogens, including UPEC^15–23^. The infection of *G. mellonella* larvae with UPEC strain CFT073 or ABU strain 83972 at 37 °C resulted in the differential expression of genes encoding TLRs, cytokine-like proteins and antimicrobial peptides (AMPs)^23^. In eukaryotes, gene expression is regulated by epigenetic mechanisms resulting in heritable phenotypes without mutation. We previously found that DNA methylation and histone acetylation were differentially regulated in larvae infected with UPEC and ABU strains, underpinning the reprogramming of innate immunity at the level of transcriptional initiation^23^.

In this follow-up study, we investigated the role of microRNAs (miRNAs), which have the potential to regulate innate immunity at the post-transcriptional level. These non-coding RNAs are 18–24 nucleotides long and are conserved in most eukaryotes. They bind to the 3′ and 5′ untranslated regions (UTRs) of target messenger RNAs (mRNAs), causing translational repression and mRNA decay^24^. They play important role in various infectious diseases, and facilitate the immune response to bacterial infection in insects^25–27^. We have constructed microarrays to analyze the expression of conserved miRNAs in *G. mellonella* larvae during infection with the entomopathogenic bacterium *Bacillus thuringiensis* and the entomopathogenic fungus *Metarhizium robertsii*^28,29^. Here, we carried out miRNA sequencing in *G. mellonella* larvae infected with UPEC strain CFT073 or ABU strain 83972 to investigate strain-dependent expression of novel and conserved miRNAs, to identify the mRNA targets of these miRNAs, and to analyze co-expression of miRNAs and their mRNA targets in infected larvae.

## Results

### Small RNA deep sequencing of *G. mellonella* larvae infected with UPEC/ABU strains

The miRNAs expressed in *G. mellonella* in response to UPEC and ABU infections were identified by high-throughput sequencing of whole-larvae samples 24 h after infection. The number of raw sequence reads was 62,511,810 for larvae infected with the UPEC strain (hereafter described as *UPEC larvae*), 53,675,182 for larvae infected with the ABU strain (hereafter described as *ABU larvae*) and 75,401,198 for the mock-injected controls. The size distribution of the trimmed, high-quality reads ranged from 17 to 30 nucleotides (Table 1) with a peak at 22 nucleotides. We identified 141 unique precursor hairpins and 257 unique mature miRNA sequences, with the greatest number detected in the UPEC larvae, followed by the control larvae and finally the ABU larvae (Table 1). Among the 257 mature miRNAs, 95 appeared to be novel, 148 were conserved and 14 included up to three mismatching nucleotides (Table S1).

**Table 1:** Length distribution of mappable reads (≥17 nt to ≤30 nt) obtained from UPEC and ABU infected *G. mellonella* deep sequencing.

Length (nt)	Number of Reads		
	CFT073	83972	Control
17	1080	304	424
18	3973	1075	1079
19	20574	5240	5460
20	61939	12931	18266
21	148923	33054	39247
22	522305	90841	111739
23	258561	43589	39245
24	186038	32528	43156
25	3906	414	539
30	0	2	0

### Expression analysis of miRNAs in *G. mellonella* larvae infected with UPEC/ABU strains

**Figure 1:**
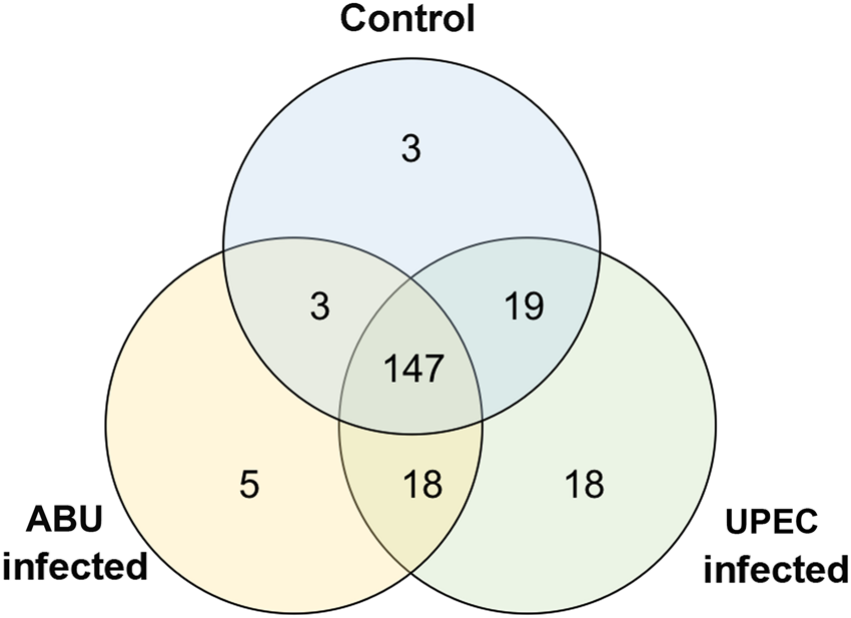
Venn diagram showing the differential expression of miRNAs in *G. mellonella* larvae infected with ABU and UPEC strains, and in mock-injected controls. The miRNA sequences were obtained following the small RNA sequencing of ABU and UPEC larvae (and mock-injected control larvae). The reads were mapped to the mature miRNAs using bwa.

We were able to classify 213 of the 257 mature miRNAs based on their comparative expression profiles in the UPEC/ABU larvae and uninfected controls. We found that 147 of the miRNAs were expressed consistently in all three groups, but 26 miRNAs were modulated in a single group and 40 miRNAs showed differential expression in pairwise comparisons (Fig. 1, Table S2). Specifically, we identified 5, 18 and 3 miRNAs that were specifically modulated in ABU larvae, UPEC larvae or controls, respectively, and we identified 3, 19 and 18 miRNAs that showed differential expression between ABU larvae and controls, between UPEC larvae and controls, and between UPEC and ABU larvae, respectively.

**Figure 2:**
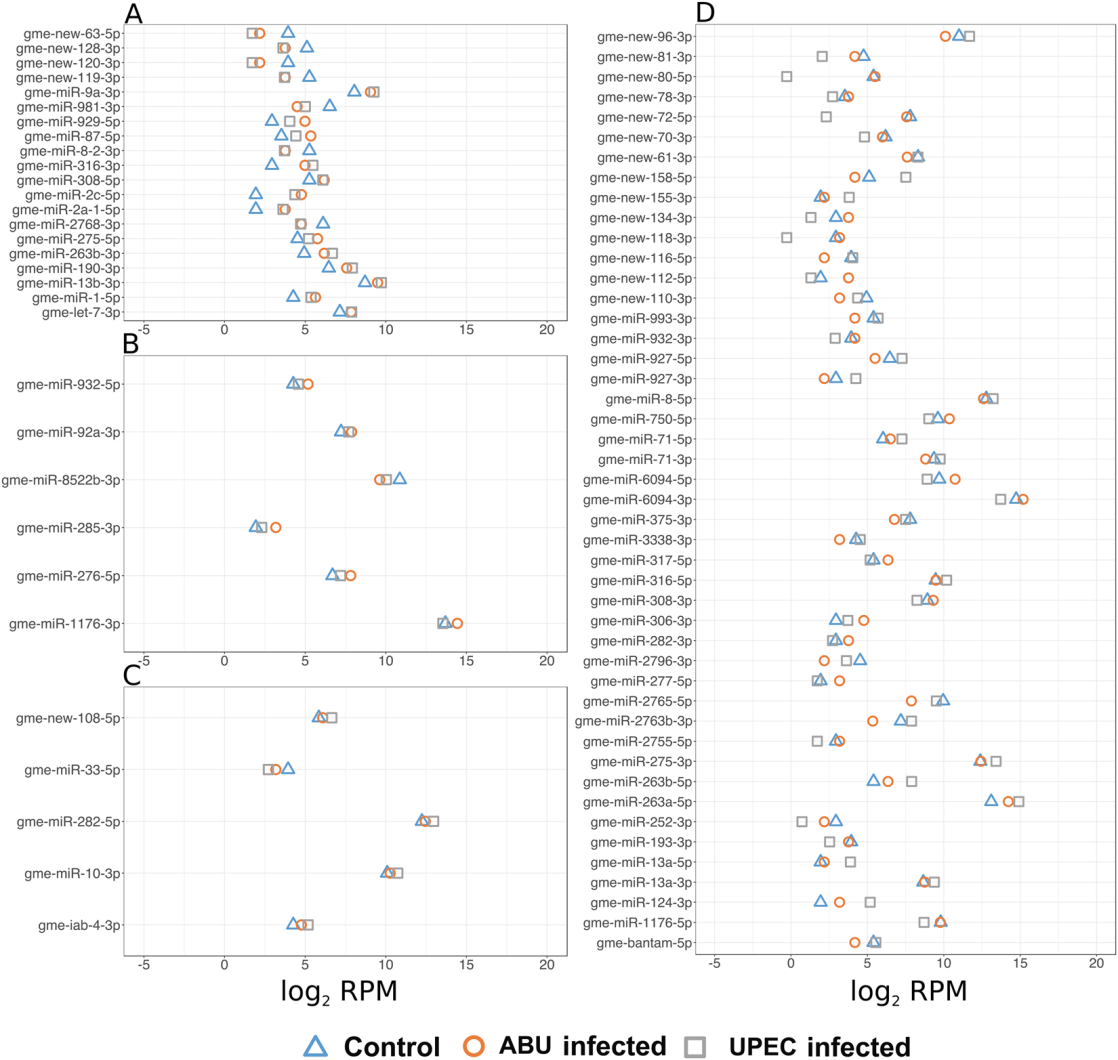
Distribution of expressed miRNAs in *G. mellonella* larvae infected with ABU and UPEC strains, and in mock-injected controls. The miRNA sequences were obtained following the small RNA sequencing of ABU and UPEC larvae (and mock-injected control larvae). (**A**–**D**) Represent significantly expressed miRNAs in ABU and UPEC infected and mock injected larvae. (**A**) miRNAs upregulated or downregulated in control larvae compared to ABU and UPEC larvae. (**B**) Majority of miRNAs upregulated in ABU larvae compared to UPEC larvae and control. (**C**) Majority of miRNAs upregulated in UPEC larvae compared to ABU larvae and control. (**D**) Majority of miRNAs upregulated or downregulated in UPEC or ABU larvae compared to control. The log expression levels were calculated in reads per million (RPM).

We selected several miRNAs for classification based on their expression profile in UPEC and ABU larvae, describing them as conserved if they could be named according to current miRBase conventions and introducing the designation *new* in the name if they were novel. We found that the novel miRNAs gme-new-63-5p, gme-new-120-3p and gme-new-119-3p were downregulated and conserved miRNAs gme-miR-316-3p, gme-miR-2c-5p, gme-miR-1-5p were upregulated in UPEC and ABU larvae compared to controls, indicating a class of miRNAs expressed generally upon bacterial infection (Fig. 2A). In contrast, the conserved miRNAs gme-miR-285-3p, gme-miR-1176-3p and gme-miR-276-5p were upregulated in ABU larvae compared to UPEC larvae and controls (Fig. 2B) whereas novel miRNAs such as gme-new-108-5p and conserved miRNAs such as gme-miR-10-3p and gme-miR-282-5p were upregulated in UPEC larvae compared to ABU larvae and controls (Fig. 2C). Similarly, the novel miRNAs gme-new-81-3p, gme-new-80-5p and gme-new-72-5p were downregulated in UPEC larvae compared to ABU larvae and controls (Fig. 2D) whereas gme-new-116-5p, gme-new-110-3p, gme-miR-993-3p and gme-miR-2765-5p were downregulated in ABU larvae compared to UPEC larvae and controls. These groupings indicated miRNAs that were specifically induced or repressed by one or other bacterial strain.

### Identification and expression analysis of miRNA targets in *G. mellonella* larvae infected with UPEC/ABU strains

**Figure 3:**
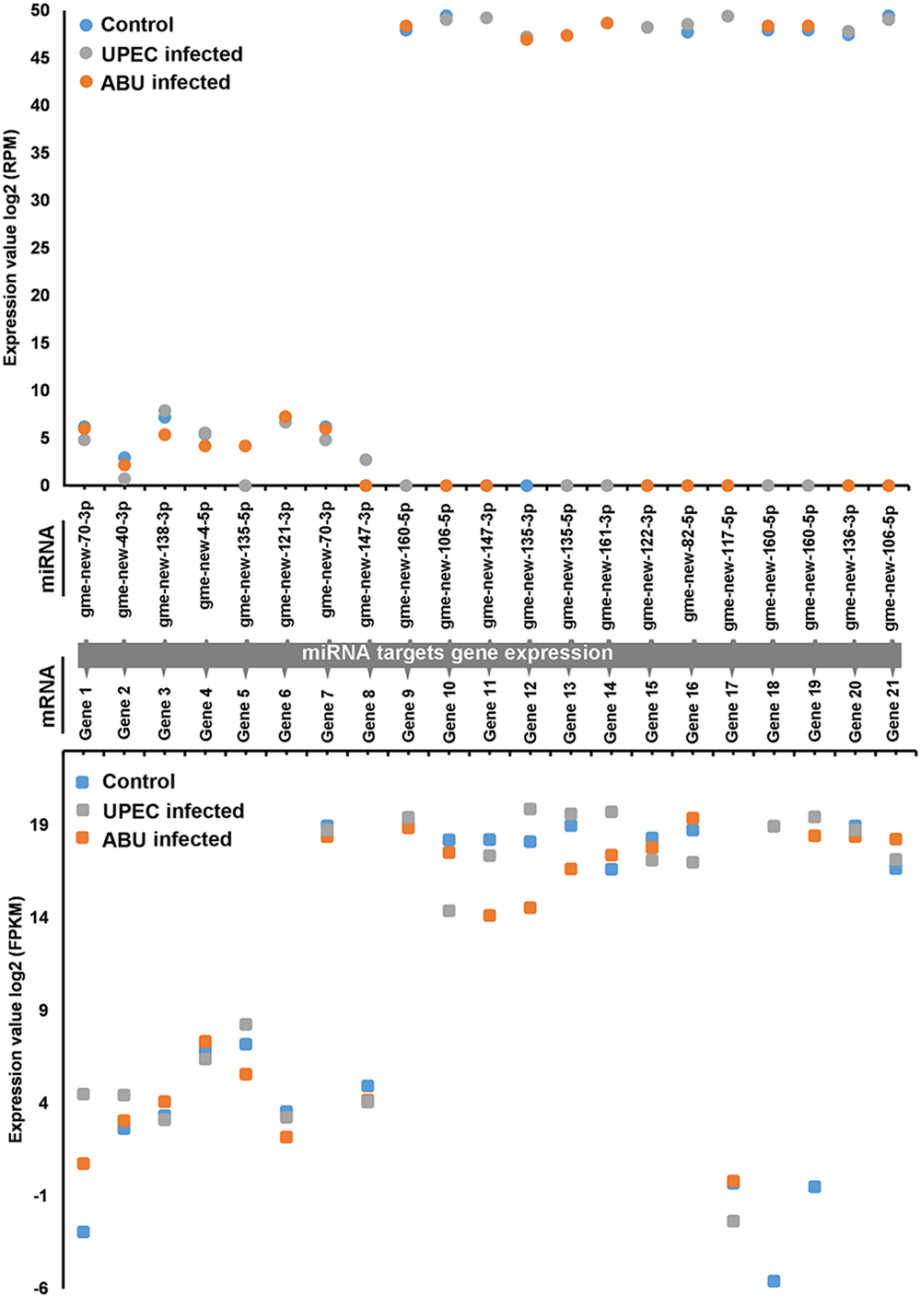
Differential expression of miRNAs and predicted target mRNAs in *G. mellonella* larvae infected with ABU and UPEC strains, and in mock-injected controls. The log expression levels of novel miRNAs identified by small RNA sequencing and their predicted mRNA targets were calculated in reads per million (RPM).

The targets of 257 mature miRNAs were predicted using our microPIECE (microRNA pipeline enhanced by CLIP experiments) pipeline^30^. This is based on argonaute-crosslinking and immunoprecipitation (AGO-CLIP) reference datasets from other insect species, allowing us to transfer the identified binding regions to orthologous transcripts in UPEC and ABU larvae. The target predictions were then mapped to conserved regions using the recently sequenced *G. mellonella* genome and transcriptome^31^ and annotated as described in the methods section. We accepted a miRNA-binding region if one of the six CLIP-seq files was positive. We identified 6979 miRNA–target interactions comprising 1898 unique target mRNAs for 257 unique miRNAs. For comparison, target prediction without CLIP-seq containment of the search space resulted in 735,748 potential miRNA–mRNA interactions. We correlated the expression of miRNAs and target mRNAs in the UPEC and ABU larvae, focusing on miRNAs targeting genes regulating innate immunity and epigenetic mechanisms in *G. mellonella*. The selected miRNAs were found to bind mRNAs encoding proteins such as AMP-binding enzyme, phosphatidylinositol 3-kinase, AMP-dependent synthetase/ligase, lysozyme, histone deacetylases (HDACs), histone acetyltransferases (HATs), and methyltransferases (Tables 2, S3). For example, gme-new-70-3p (target gene 1), gme-new-40-3p (target gene 2), gme-new-135-5p (target gene 13) and gme-new-160-5p (target gene 19) were downregulated in UPEC larvae compared to ABU larvae and controls, and their corresponding target mRNAs encoding AMP-binding enzyme, phosphatidylinositol 3-kinase, AGC-kinase C-terminal domain, and lipopolysaccharide-induced tumor necrosis alpha factor (LITAF) were upregulated (Fig. 3). Conserved and novel miRNAs targeting mRNAs encoding immunity-related proteins such as TNF-8-like or zf-LITAF-like were either upregulated (gme-miR-274-3p and gme-miR-8-5p) or downregulated (gme-new-135-3p, gme-new-161-3p and gme-new-160-5p) in UPEC larvae compared to ABU larvae, or uniformly expressed (gme-miR-124-5p, gme-miR-2a-3p and gme-miR-13b-3p) (Fig. S1). Furthermore, miRNAs targeting mRNAs encoding invertebrate-type lysozyme were either upregulated (gme-miR-263a-5p) or downregulated (gme-new-135-5p and gme-miR-263b-5p) in UPEC larvae compared to ABU larvae, or uniformly expressed (gme-miR-2a-2-5p) (Fig. S2). Novel miRNAs targeting mRNAs encoding linear gramicidin synthase subunit D, long-chain fatty acid–CoA ligase and Ras guanine-nucleotide exchange factor were either upregulated (gme-new-138-3p, gme-new-54-3p and gme-new-4-5p) or downregulated (gme-new-40-3p, gme-new-30-3p and gme-new-70-3p) in UPEC larvae compared to ABU larvae, or uniformly expressed (gme-new-72-3p) (Fig. S3).

**Table 2:** Annotation of miRNA targets.

miRNA	Target mRNA	mRNA Annotation
gme-new-70-3p	Gene 1	AMP-binding enzyme
gme-new-40-3p	Gene 2	Phosphatidylinositol 3-kinase, C2 domain
gme-new-138-3p	Gene 3	AMP-dependent synthetase/ligase
gme-new-4-5p	Gene 4	AMP-dependent synthetase/ligase
gme-new-135-5p	Gene 5	Invertebrate-type lysozyme
gme-new-121-3p	Gene 6	Acetyltransferase (GNAT) family
gme-new-70-3p	Gene 7	Aldolase-type TIM barrel
gme-new-147-3p	Gene 8	Histone deacetylase superfamily
gme-new-160-5p	Gene 9	Ubiquitin-activating enzyme
gme-new-106-5p	Gene 10	S-adenosyl-L-methionine-dependent methyltransferase
gme-new-147-3p	Gene 11	Histone deacetylase superfamily
gme-new-135-3p	Gene 12	AMP-dependent synthetase
gme-new-135-5p	Gene 13	AGC-kinase C-terminal domain
gme-new-161-3p	Gene 14	AMP-dependent synthetase/ligase
gme-new-122-3p	Gene 15	Ubiquitin carboxyl-terminal hydrolase superfamily
gme-new-82-5p	Gene 16	HECT, E3 ligase catalytic domain
gme-new-117-5p	Gene 17	Ubiquitin-like domain superfamily
gme-new-160-5p	Gene 18	LPS-induced tumor necrosis factor alpha factor
gme-new-160-5p	Gene 19	LITAF domain containing protein
gme-new-136-3p	Gene 20	Histone-lysine N-methyltransferase
gme-new-106-5p	Gene 21	Acetyltransferase (GNAT) domain

Similarly, gme-new-106-5p (target gene 10), gme-new-147-3p (target gene 11), gme-new-122-3p (target gene 15), gme-new-82-5p (target gene 16), gme-new-117-5p (target gene 17) and gme-new-136-3p (target gene 20) were downregulated in ABU larvae compared to UPEC larvae and controls, and their corresponding target mRNAs encoding methyltransferases, HDACs and hydrolases were upregulated (Fig. 3). The miRNAs targeting mRNAs encoding HATs were either upregulated (gme-miR-184-5p and gme-miR-13a-3p) or downregulated (gme-new-70-3p and gme-new-135-3p) in UPEC larvae compared to ABU larvae, or uniformly expressed (gme-miR-970-3p and gme-new-108-3p) (Fig. S4). The miRNAs targeting mRNAs encoding HDACs were either upregulated (gme-new-147-3p, gme-miR-71-3p and gme-miR-316-5p) or downregulated (gme-new-134-3p, gme-new-81-5p and gme-new-112-5p) in UPEC larvae compared to ABU larvae, or uniformly expressed (gme-miR-2766-3p, gme-new-80-3p and gme-miR-33-3p) (Fig. S5). Novel miRNAs targeting mRNAs encoding methyltransferases were either upregulated (gme-new-136-3p, gme-new-149-3p and gme-new-61-5p), or downregulated (gme-new-160-5p, gme-new-135-3p and gme-new-123-3p) in UPEC larvae compared to ABU larvae, or uniformly expressed (gme-new-139-5p, gme-new-128-5p and gme-new108-3p) (Fig. S6). The numbers of miRNA targets identified by microPIECE in Fig. 3 were validated by RNAhybrid (3′ UTR_Fly) and RNA22 (sensitivity 63%, specificity 61%, minimal number of paired-up bases in heteroduplexes 10, max fold energy -5). With RNAhybrid, we confirmed all 21 predicted targets, whereas with RNA22 v2 16 of 21 microRNA-mRNA pairs were validated (Table 3).

**Table 3:** Validation of miRNA target prediction by microPIECE from Table 2.

miRNA – target mRNA	miRanda	RNAhybrid	RNA22
gme-new-70-3p – Gene 1	●	●	○
gme-new-40-3p – Gene 2	●	●	●
gme-new-138-3p – Gene 3	●	●	●
gme-new-4-5p – Gene 4	●	●	●
gme-new-135-5p – Gene 5	●	●	●
gme-new-121-3p – Gene 6	●	●	●
gme-new-70-3p – Gene 7	●	●	○
gme-new-147-3p – Gene 8	●	●	●
gme-new-160-5p – Gene 9	●	●	●
gme-new-106-5p – Gene 10	●	●	○
gme-new-147-3p – Gene 11	●	●	●
gme-new-135-3p – Gene 12	●	●	●
gme-new-135-5p – Gene 13	●	●	●
gme-new-161-3p – Gene 14	●	●	●
gme-new-122-3p – Gene 15	●	●	○
gme-new-82-5p – Gene 16	●	●	●
gme-new-117-5p – Gene 17	●	●	●
gme-new-160-5p – Gene 18	●	●	●
gme-new-160-5p – Gene 19	●	●	●
gme-new-136-3p – Gene 20	●	●	●
gme-new-106-5p – Gene 21	●	●	○
●	**←** Successful prediction	No prediction **→**	○

**Figure 4:**
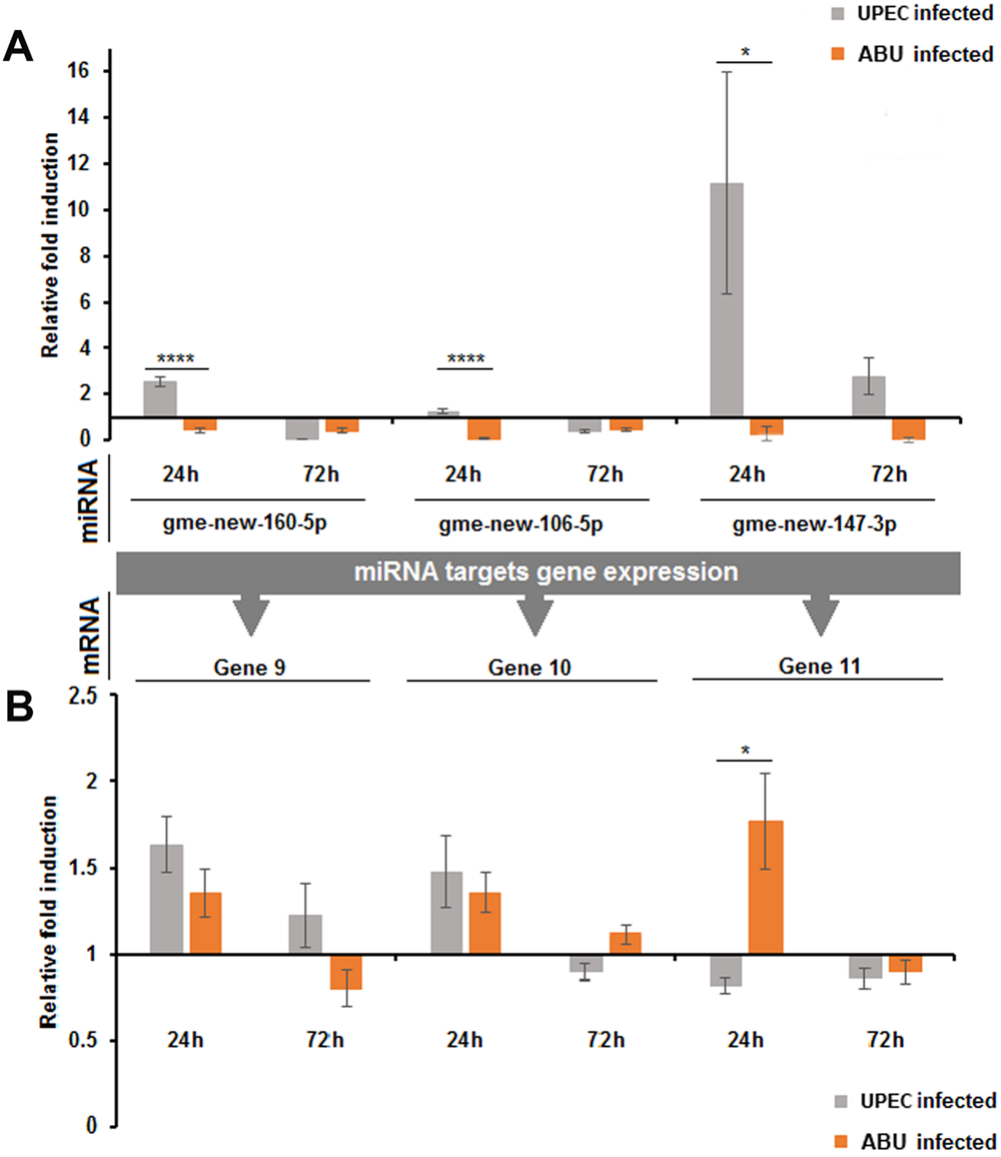
Differential expression of selected miRNAs and target mRNAs by RT-PCR in *G. mellonella* larvae infected with ABU and UPEC strains, and in mock-injected controls. The novel miRNA sequences were obtained from small RNA sequencing and their predicted mRNA targets were validated by RT-PCR to confirm differential expression: (**A**) gme-new-160-5p, gme-new-106-5p, gme-new-147-3p; (**B**) gene 9, gene 10, gene 11. The relative fold differences indicated for the miRNAs and mRNAs are normalized against gme-miR-133 and elongation factor 1, respectively, as the internal reference controls (****p < 0.0001, *p < 0.05).

The expression of miRNA candidates and their mRNA targets was further experimentally verified by RT-PCR. We selected gme-new-160-5p, gme-new-106-5p, gme-new-147-3p, gme-miR-929-5p, gme-miR-932-5p and gme-miR-let-7-5p because of their relatively high expression levels in UPEC/ABU larvae and confirmed their upregulation (gme-new-106-5p and gme-new-147-3p) or downregulation (gme-miR-929-5p) in UPEC larvae relative to ABU larvae 24 h after infection, as predicted by miRNA sequencing (Figs. 4A, S7). No differences in the expression levels of these miRNAs were observed 72 h after infection. The upregulation of gme-new-147-3p in UPEC larvae compared to ABU larvae resulted in the downregulation of its predicted target mRNA, for gene 11 (Fig. 4B).

## Discussion

Host susceptibility and innate immunity-related gene expression help determine the outcome of infections caused by UPEC and ABU strains in the urinary tract. In this study, we used the surrogate insect host *G. mellonella* to show that the differential innate immune response to UPEC and ABU infections is regulated at least in part by non-coding miRNAs. Following miRNA sequencing in ABU and UPEC larvae, we analyzed the strain-dependent expression of novel and conserved miRNAs, and using our microPIECE pipeline based on AGO-CLIP reference datasets from other insect species we predicted miRNA targets^30^. We found that ABU and UPEC infections can trigger the expression of novel and conserved miRNAs to modulate the expression of innate immunity-related genes (Figs. 3, S1–S3). ABU infection induces miRNAs that suppress genes related to cell signaling and innate immunity. For example, the expression of gme-new-160-5p inhibits LITAF like immunity related proteins in larvae and reduces the lipopolysaccharide-induced innate immune activation, resulting in improved host survival^32,33^. The post-transcriptional suppression of LITAF may thus favor the long-term survival of ABU in larvae by attenuating the immune response, whereas the expression of LITAF in UPEC larvae encourages strong immune response^23^. Inhibition of LITAF provides resistance to systemic *E. coli* LPS-induced lethality in mammals. Thus, LITAF is a promising therapeutic target for the treatment of TNF-mediated inflammatory diseases, and we identified a miRNA in *G. mellonella* that inhibits its expression^33^. ABU and UPEC infections also modulate miRNAs that affect the production of AMPs. For example, UPEC infections induce the expression of gme-138-3p, gme-new-4-5p and gme-new-135-3p while suppressing gme-new-70-3p and gme-new-161-3p, which has the net effect of increasing the synthesis of the most potent AMPs (lysozymes, cecropins, gloverin, galiomycin and moricin), whereas weaker AMPs (anionic peptides, apolipophoricin) are induced following ABU infection^23^. ABU *E. coli* strain 83972 differs from UPEC strain CFT073 in terms of virulence gene expression^6^. Colonization and long-term survival of ABU *E. coli* strain 83972 in *G. mellonella* larvae and most likely also in the human urinary tract is achieved by miRNA-mediated suppression of gene expression constituting strong antimicrobial response. On the other hand, UPEC infection provokes expression of these AMPs by downregulating miRNAs that specifically inhibit their expression. The expression of miRNAs targeting AMP synthesis has also been shown following infection in the insect host *Plutella xylostella*, and in some human diseases^34,35^. We used the *G. mellonella* system to discover new miRNAs that target innate immune related proteins important for UPEC infection implicating scope for therapeutic application to treat UTI in humans.

Several human pathogens can manipulate host cell antimicrobial responses and evade the immune system by influencing epigenetic mechanisms such as histone acetylation and DNA/RNA methylation^36^. Qualitative and quantitative differences in the responses to UPEC and ABU strains in *G. mellonella* are also epigenetically regulated in this manner^23^. While many target genes of miRNAs are known, even less information exists as to how miRNAs cooperate with histone acetylation and DNA methylation in the context of host-pathogen interaction. There is limited evidence suggesting that negative correlation between the expression of miRNAs and HDACs or HATs has been associated with infection by human pathogens^37^. Interestingly, our analysis of miRNA targets revealed that both novel and conserved miRNAs can target genes encoding methyltransferases, HATs and HDACs, which are key regulators of DNA methylation and histone modifications. In UPEC larvae, the induction of miRNAs gme-new-106-5p, gme-new-184-5p and gme-new-13a-3p correlated with the downregulation of target genes encoding HATs, which acetylate histones to form open chromatin that favors the expression of immunity-relate genes. In mammals, HATs are degraded by a zinc-dependent metalloproteinase from enteropathogenic and enterohemorrhagic *E. coli* in order to dampen inflammatory responses^38,39^. We suggest that UPEC also follow an alternative strategy of miRNA-mediated suppression of HAT expression in an infected host. The activity of HATs is also opposed by HDACs, which deacetylate histones and form condensed chromatin that suppresses gene expression. The HDAC sap18 subunit was downregulated in ABU larvae but upregulated in UPEC larvae and here we identified a miRNA (gme-new-134-3p) that targets the mRNA encoding this protein subunit^21^. The induction of this novel miRNA in ABU larvae suggests a post-transcriptional mechanism for the suppression of HDAC sap18. The novel miRNA gme-new-106-5p was upregulated in UPEC larvae, and this targets the mRNA for S-adenosyl-L-methionine-dependent methyltransferase, which is a methyl donor for DNA methyltransferases. We identified miRNAs that target methyltransferase mRNAs in UPEC and ABU larvae, but surprisingly none of them were the DNA methyltransferases responsible for epigenetic regulation. However, insect genomes are sparsely methylated compared to mammals, and the role of DNA methylation in innate immunity is not well understood. The lack of miRNAs targeting mRNAs encoding maintenance or *de novo* methyltransferases in UPEC and ABU larvae may indicate the limited significance of DNA methyltransferases in the regulation of innate immunity in *G. mellonella*^40^.

In addition to the strain-specific regulation of miRNAs, we also identified a large number of miRNA candidates that were commonly modulated in UPEC and ABU larvae. The majority of these miRNAs (such as mir-8 family) are conserved among other eukaryotes, for example let-7 and mir-124 have homologous targets in *G. mellonella* and humans. Gme-let-7-5p targets the HAT KAT2A, and both gme-miR-283-5p and gme-miR-33-3p target HDAC8, indicating the existence of cross-talk between miRNAs and other epigenetic mechanisms that regulate chromatin structure and gene expression^41^.

## Conclusion

Here we show that UPEC strain CFT073 and ABU strain 83972 trigger the modulation of host miRNAs in *G. mellonella* to epigenetically regulate the innate immune response. Many novel miRNAs showed strain-dependent expression in the infected larvae, whereas others were modulated similarly regardless of the infection status or in a similar manner during both infections. We argue that miRNAs determine the different pathogenic potential of the ABU and UPEC strains in *G. mellonella*, and may regulate different behavior of these strains in the human urinary tract. Taken together, our results emphasize the importance of *G. mellonella* miRNAs in the regulation of host innate immunity to distinguish between pathogenic and commensal-like *E. coli* strains.

## Methods

### Bacterial strains, insects, and culture media

Cultures of UPEC strain CFT073 and ABU strain 83972 were maintained aerobically in lysogeny broth (LB) at 37 °C and on LB agar plates (Carl Roth, Karlsruhe, Germany). For long-term storage, bacteria were frozen at −80 °C in LB supplemented with 30% (v/v) glycerol. *G. mellonella* larvae were obtained from Fauna Topics Zoobedarf Zucht und Handels GmbH, Marbach am Neckar, Germany. The larvae were reared on an artificial diet (22% maize meal, 22% wheat germ, 11% dry yeast, 17.5% beeswax, 11% honey and 11% glycerin) at 32 °C in darkness. Larvae at their sixth instar stage, each weighing 250–350 mg, were used in all experiments.

### *G. mellonella* injection

Injection experiments were carried out using logarithmic growth-phase bacterial cultures in 10 ml LB. The bacteria were washed and serially diluted in 0.9% NaCl, and 10-μl aliquots (10^5^ colony forming units/ml) were injected into larvae through the left proleg using 1-ml disposable syringes and 20-mm 0.4 gauge needles mounted on a microapplicator, as previously described^42^. Mock injections with an empty needle were carried out as controls. Larvae were considered dead after incubation at 37 °C when they showed no movement in response to touch.

### Small RNA isolation, library construction, sequencing and analysis

We used the following criteria to design experiments and selected miRNA sequencing techniques, to minimize sequencing error rates and limitations for not performing cost-intensive multiple miRNA sequencing experiments. First, considering the importance of biological variations in the identification of conserved and highly expressed miRNA, total RNA samples were extracted using the miRNeasy Mini Kit (Qiagen, Hilden, Germany) and pooled from three independent biological experiments comprising five larvae per experiment. Sequencing of miRNAs was performed by LC Sciences (Houston, Texas, USA). Briefly, total RNA was analyzed using a Nandrop spectrophotometer (Thermo Fischer Scientific, Waltham, Massachusetts, USA) and a 2100 Bioanalyzer (Agilent Technologies, Santa Clara, California, USA) and samples with an RNA integrity number (RIN) > 7 as well as 260/280 and 260/230 absorbance ratios > 2.0 were used for cDNA library preparation based on the TruSeq Small RNA Sample Preparation Protocol (Illumina, San Diego, California, USA). Next, the cDNA libraries were purified and sequenced on an Illumina HiSeq. 2500 in the 50-bp single-end configuration, which has the least error rate (below 0.1%) compared to other sequencing techniques. *G. mellonella* miRNAs were identified using miRDeep2^43^ v2.0.0.8 to screen the recently published *G. mellonella* genome sequence against miRbase^44^ release 22. We designated miRNAs from all animals including related insect species *Bombyx mori*, *Manduca sexta*, *Plutella xylostella*, *Spodoptera frugiperda* (order Lepidoptera), *Aedes aegypti*, *Anopheles gambiae*, *Tribolium castaneum*, *Drosophila melanogaster* (order Diptera), *Apis mellifera*, *Nasonia vitripennis* (order Hymenoptera), and *Acyrthosiphon pisum* (order Homoptera) for the purpose of miRNAs identification. The miRNA hits were temporarily numbered in ascending order and identical mature sequences were merged to reduce redundancy. The miRNAs with up to three nucleotide mismatches were renamed according to the best match from miRBase. Next, to reduce false positives, we discarded all reads that mapped to rRNAs from further analysis. Briefly, reads with a minimal length of 17 nucleotides were trimmed using cutadapt v1.8.3 and rRNA sequences were filtered out by screening against the Silva rRNA database^45,46^. Mature miRNAs were mapped using bwa v0.7.10-r789 and we calculated the expression level in reads per million (RPM)^46^,^47^. We used Deseq v1.32.0 to compute an indicative fold change between samples^47^,^48^ and the stem loop structures of 37 novel miRNAs were determined (Table S4). For the expression calculation, we allowed one measured miRNA read, because adapter ligations in library preparation steps, as well as unwanted intra- and inter-RNA bindings of miRNAs can disfavor certain mature miRNAs and may lead to underrepresented sequencing results. We considered results with a fold change of ≤0.5 and ≥1.5 as differentially expressed between two groups of larvae.

### Annotation of *G. mellonella* transcriptome and miRNA target prediction

For miRNA target prediction, the publicly available paired-end RNA sequencing libraries from UPEC and ABU infected *G. mellonella* larvae (SRX2727976 and SRX2727977) were compared with non-infected larvae (SRX371340). We then aligned the reads from the transcriptome sequencing against the recently sequenced *G. mellonella* genome (ASM258982v1)^31^ using HiSat2^49^ v2.1.0 and transformed and sorted the results using samtools^49^ v1.6. Assembly was carried out using cufflinks v2.2.1 with standard parameters^50^ and TransDecoder v5.0.2 analysis for the annotation of different isoforms^51^. The analysis was enhanced with a BlastP v2.6.0 + search against SwissProt^52^ and a hmmer^53^ search against Pfam-A with standard parameters^53^. The assembly was evaluated using rnaQUAST^54^,^55^. We then annotated the transcripts using InterproScan v5.27-66.0.

Targets were predicted using the microPIECE pipeline^30^, which uses AGO-CLIP libraries from other species and performs a trimming, mapping and peak calling on the datasets, like a typical CLIP analysis workflow. Briefly, the microPIECE takes the AGO-CLIP data from a species A and transfers it to a species B. That means it transfer the verified miRNA binding region of a mRNA to the homologous transcript of another species. Given a set of miRNAs from species B the pipeline then predicts their targets on the transferred CLIP regions with a much lower false-positive rate, since it relies on evolutionary conserved and experimentally verified binding regions. Identification of orthologous proteins and mapping of the signaling regions of miRNA binding sites to the orthologous transcripts in the species of interest were computed and used for target predictions with miRanda v3.3a. The transfer of known miRNA binding sites was achieved using a set of six publicly available *A. aegypti* AGO-CLIP libraries, which is the only AGO-CLIP dataset available in the clade of insects^48^,^56^. From those six libraries, there are three replicates for two conditions. The resulting mRNA target sites that we have identified by using *A. aegypti* as AGO-CLIP data donor for *G. mellonella* are expected to be relatively small than a more closely related insect species, but this technique ensures that at least those target sites are already present between *A. aegypti* and *G. mellonella*. In addition to the miRanda tool, we used the prediction algorithms RNAhybrid and RNA22 v2 to validate the miRNA target prediction by microPIECE. We followed union over intersection when combining several tools for miRNA-mRNA predictions^57^.

### RT-PCR analysis

Relative miRNA and mRNA expression levels were determined by RT-PCR as previously described^25^. For the analysis of miRNAs, cDNA was synthesized using the miScript II miRNA first-strand synthesis and qPCR kit (Qiagen, Hilden, Germany). Small RNA-enriched total RNA was reverse transcribed using miScript HiSpec buffer, modified oligo-dT primers with 3′ degenerate anchors and 5′ universal tag sequence for the specific synthesis of mature miRNAs. The combination of polyadenylation and the universal tag ensures that miScript primer assays do not detect genomic DNA. Primers for the selected miRNAs were designed using the miScript miRNA product design webpage (Qiagen, Hilden, Germany). Candidate miRNA expression levels were normalized against gme-miR-133, which showed uniform expression across all samples. Real-time RT-PCR was carried out using the CFX 96 Mx3000P system (Bio-Rad, Hercules, California, USA), starting with a 15-min incubation at 95 °C to activate the Hot Start Polymerase followed by 40 cycles at 94 °C for 15 s, 55 °C for 30 s and 70 °C for 30 s. The following miRNA sequences were used for primer design: gme-new-160-5p, 5′- GTC ATT CAG CCT GCC AGC ATT GCT-3′; gme-new-106-5p, 5′-CCT TGT CAT TCT TCT TGC CCA GT-3′; gme-new-147-3p, 5′-ATT TGG TTC TCT CTA ATA GCA AT-3′; gme-miR-929-5p, 5′-AAA TTG ACT CTA GTA GGG AGT-3′; gme-miR-932-5p, 5′-TCA ATT CCG TAG TGC ATT GCA GT-3′; gme-miR-let-7-5p, 5′-TGA GGT AGT AGG TTG TAT AG -3′. The control miRNA sequence was gme-miR-133, 5′-AAG TTT TCC GTG ACG ATA TAA GGG GGC TCC-3′. The amplification of specific target mRNAs by RT-PCR was carried out as previously described^17^ using the following primer sequences: Gene 9-fwd 5′- CTA CAC TCG TCG CAG CAC AT-3′ and -rev 5′-GTG TTA CGG TGC ATT GTT GG-3′; Gene 10-fwd 5′-CAC CGC CTG GTA AAG AAC TC-3′ and -rev 5′-CCA TTT GAA TCC CAA GTG GA-3′; Gene 11-fwd 5′-GGC CGA TGT GTG GAG TTA GT-3′; and -rev 5′-TGC TGG GTG ATA TGT GCA GT-3′; and the housekeeping gene elongation factor 1-fwd 5′-ATG GTT GCA AAG CTG AAA CT-3′ and -rev 5′-TCC CGT GTT GAG TCA AAT TA-3′.

## Data analysis

Data in Figs. 3 and 4 were analyzed using Microsoft Excel 2013 (Microsoft Corp., Redmond, Washington, USA). All experiments except small RNA sequencing were performed a minimum of three times. Significant differences between pairs of values were compared using one-way analysis of variance and the Holm-Šídák test.

## Supplementary information

Supplementary information.

Supplementary table S1.

## Data Availability

The small RNA sequencing data is available at NCBI via the accession number GSE123965. All data are accessible in the Supplementary Information.
