# Supplementary material for: MicroRNAs regulate innate immunity against uropathogenic and commensal-like Escherichia coli infections in the surrogate insect model Galleria mellonella
Source: Sci Rep. 2020 Feb 13;10:2570. doi: 10.1038/s41598-020-59407-3 (PMC7018962; doi:10.1038/s41598-020-59407-3)
Supplement: Supplementary file 1 — Supplementary information. [file 41598_2020_59407_MOESM1_ESM.docx]

# **MicroRNAs regulate innate immunity against uropathogenic and commensal-like *Escherichia* coli infections in the surrogate insect model *Galleria mellonella***

# Krishnendu Mukherjee^1, 3†^, Daniel Amsel^1†^, Miriam Kalsy^1^, Andre Billion^1^, Ulrich Dobrindt^3^, Andreas Vilcinskas^2#^

^1^Fraunhofer Institute for Molecular Biology and Applied Ecology, Department of Bioresources, Winchester Str. 2, 35394 Giessen, Germany

^2^Institute for Insect Biotechnology, Justus Liebig University, Heinrich-Buff-Ring 26-32, 35392 Giessen, Germany

^3^Institute of Hygiene, University of Münster, Mendelstrasse 7, 48149 Münster, Germany

#corresponding author

^†^Authors Contributed Equally

Email: Andreas.Vilcinskas@agrar.uni-giessen.de


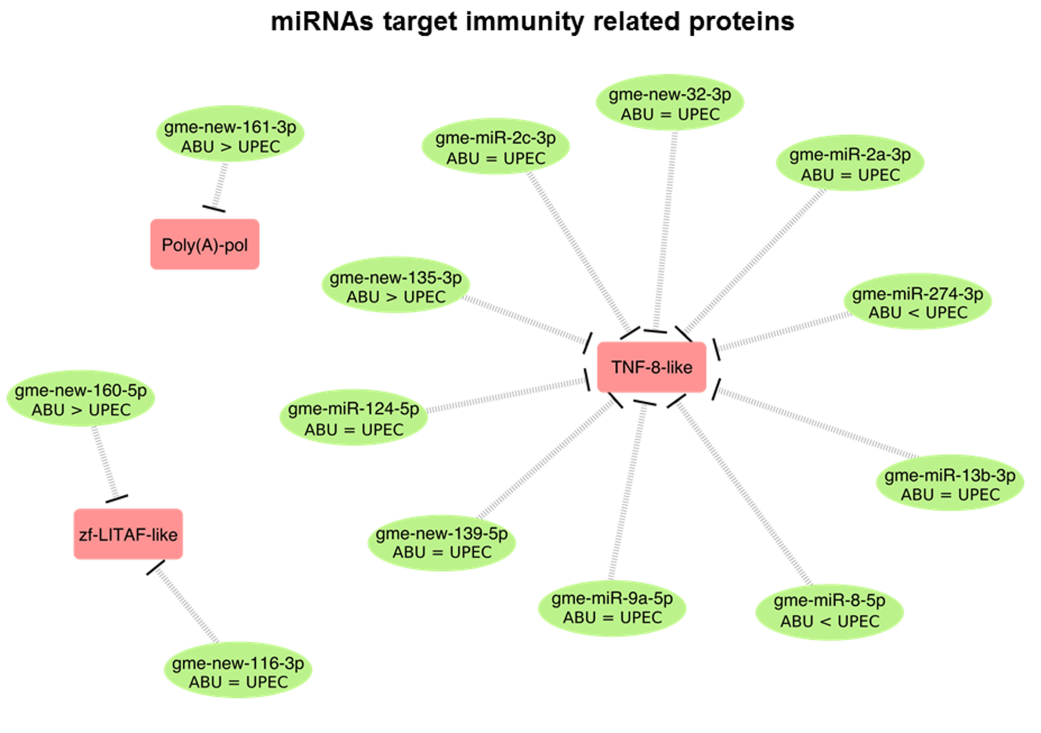


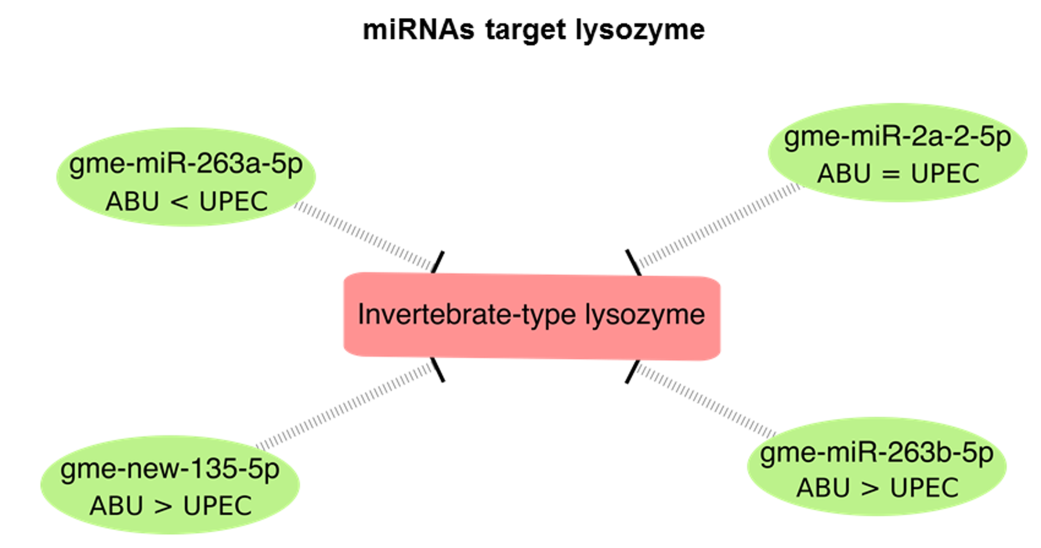
Supplementary Fig. S1. **The immunity related mRNAs as target of miRNAs in ABU and UPEC infected *G. mellonella* larvae.** The network diagram generated with Cytoscape shows mRNAs related to innate immunity related proteins targeted by miRNAs in ABU and UPEC infected larvae. Target mRNAs are represented in different colors to maintain contrast with the miRNAs. Connecting lines (edges) are used to indicate miRNA targets identified in this study by consulting Gene Ontology terms related to *G. mellonella* genome and transcriptome sequences. ABU<UPEC means miRNA upregulation in UPEC compared to ABU, ABU>UPEC means miRNA upregulation in ABU compared to UPEC, and ABU=UPEC means no difference in miRNA expression.

Supplementary Fig. 2. **The lysozyme mRNA as targets of miRNAs in ABU and UPEC infected *G. mellonella* larvae.** The network diagram generated with Cytoscape shows mRNAs encoding lysozyme targeted by miRNAs in ABU and UPEC infected larvae. Target mRNAs are represented in different colors to maintain contrast with the miRNAs. Connecting lines (edges) are used to indicate miRNA targets identified in this study by consulting Gene Ontology terms related to *G. mellonella* genome and transcriptome sequences. ABU<UPEC means miRNA upregulation in UPEC compared to ABU, ABU>UPEC means miRNA upregulation in ABU compared to UPEC, and ABU=UPEC means no difference in miRNA expression.


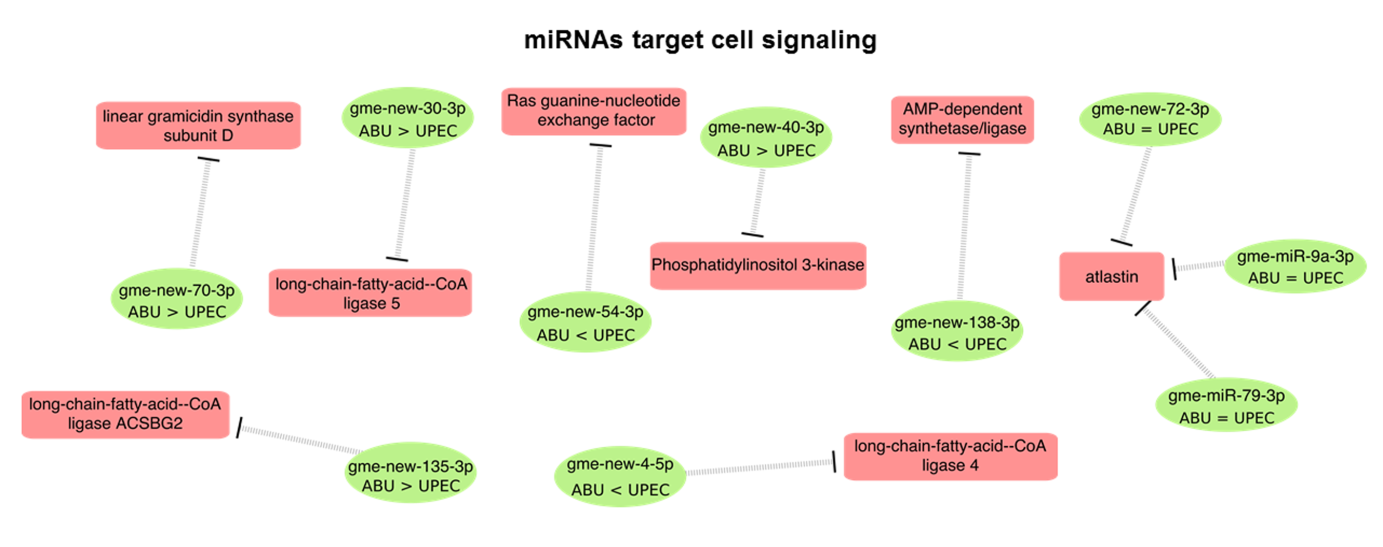


Supplementary Fig. S3. **The mRNAs related to cell signaling as targets of miRNAs in ABU and UPEC infected *G. mellonella* larvae.** The network diagram generated with Cytoscape shows mRNAs related to cell signaling targeted by miRNAs in ABU and UPEC infected larvae. Target mRNAs are represented in different colors to maintain contrast with the miRNAs. Connecting lines (edges) are used to indicate miRNA targets identified in this study by consulting Gene Ontology terms related to *G. mellonella* genome and transcriptome sequences. ABU<UPEC means miRNA upregulation in UPEC compared to ABU, ABU>UPEC means miRNA upregulation in ABU compared to UPEC, and ABU=UPEC means no difference in miRNA expression.


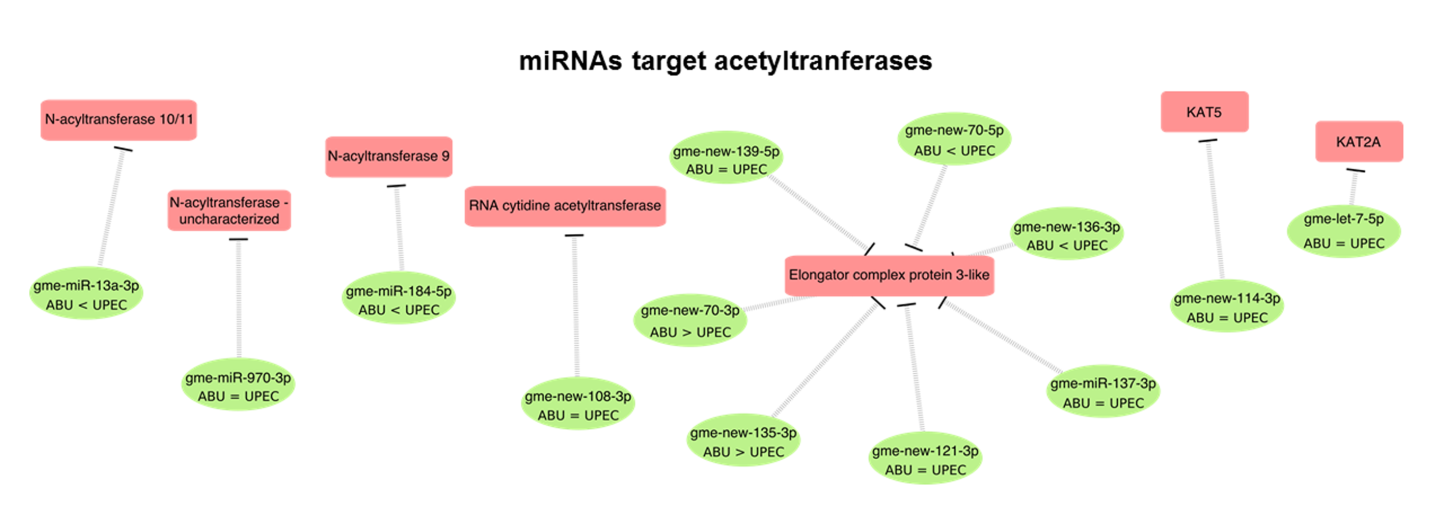


Supplementary Fig. S4. **The enzyme acetyltransferase mRNAs as targets of miRNAs in ABU and UPEC infected *G. mellonella* larvae.** The network diagram generated with Cytoscape shows mRNAs encoding acetyltranferases targeted by miRNAs in ABU and UPEC infected larvae. Target mRNAs are represented in different colors to maintain contrast with the miRNAs. Connecting lines (edges) are used to indicate miRNA targets identified in this study by consulting Gene Ontology terms related to *G. mellonella* genome and transcriptome sequences. ABU<UPEC means miRNA upregulation in UPEC compared to ABU, ABU>UPEC means miRNA upregulation in ABU compared to UPEC, and ABU=UPEC means no difference in miRNA expression.


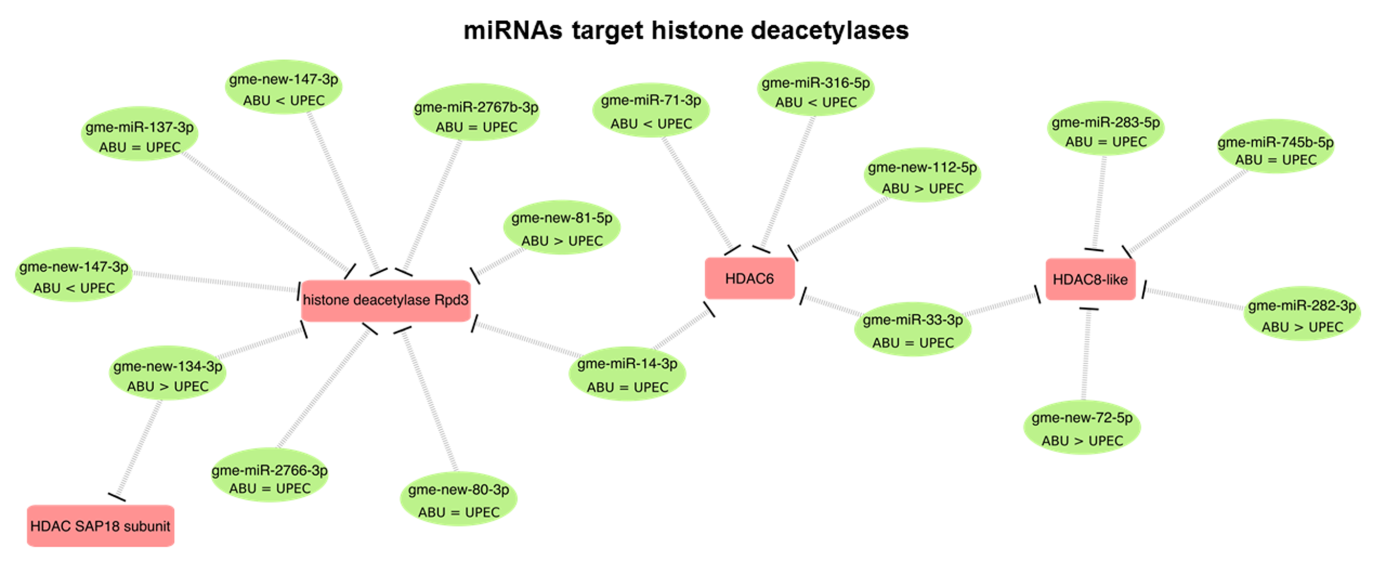


Supplementary Fig. S5. **The enzyme histone deacetylases mRNAs as targets of miRNAs in ABU and UPEC infected *G. mellonella* larvae.** The network diagram generated with Cytoscape shows mRNAs encoding histone deacetylases targeted by miRNAs in ABU and UPEC infected larvae. Target mRNAs are represented in different colors to maintain contrast with the miRNAs. Connecting lines (edges) are used to indicate miRNA targets identified in this study by consulting Gene Ontology terms related to *G. mellonella* genome and transcriptome sequences. ABU<UPEC means miRNA upregulation in UPEC compared to ABU, ABU>UPEC means miRNA upregulation in ABU compared to UPEC, and ABU=UPEC means no difference in miRNA expression.


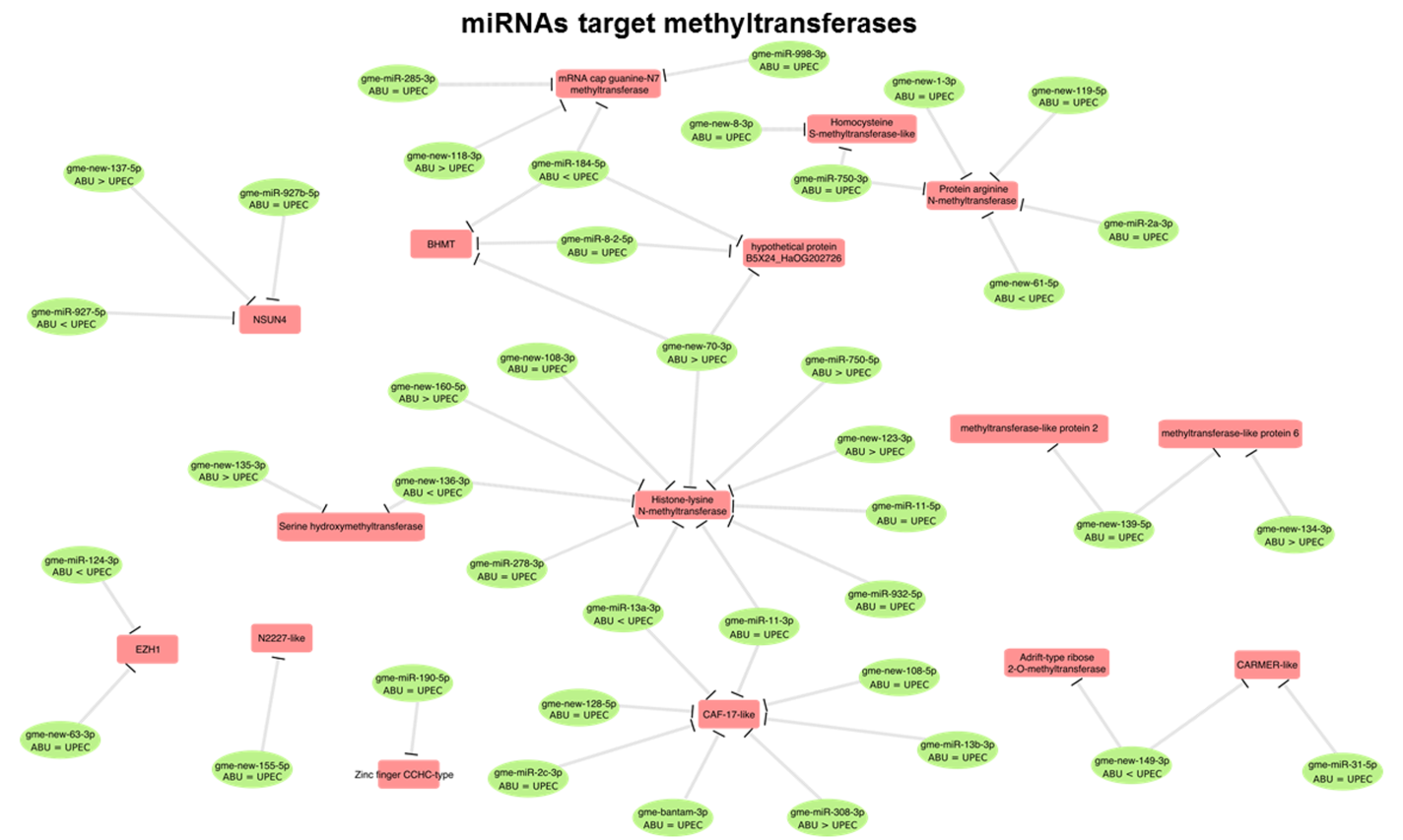


Supplementary Fig. S6. **The enzyme methyltransferases mRNAs as targets of miRNAs in ABU and UPEC infected *G. mellonella* larvae.** The network diagram generated with Cytoscape shows mRNAs encoding methyltransferases targeted by miRNAs in ABU and UPEC infected larvae. Target mRNAs are represented in different colors to maintain contrast with the miRNAs. Connecting lines (edges) are used to indicate miRNA targets identified in this study by consulting Gene Ontology terms related to *G. mellonella* genome and transcriptome sequences. ABU<UPEC means miRNA upregulation in UPEC compared to ABU, ABU>UPEC means miRNA upregulation in ABU compared to UPEC, and ABU=UPEC means no difference in miRNA expression.


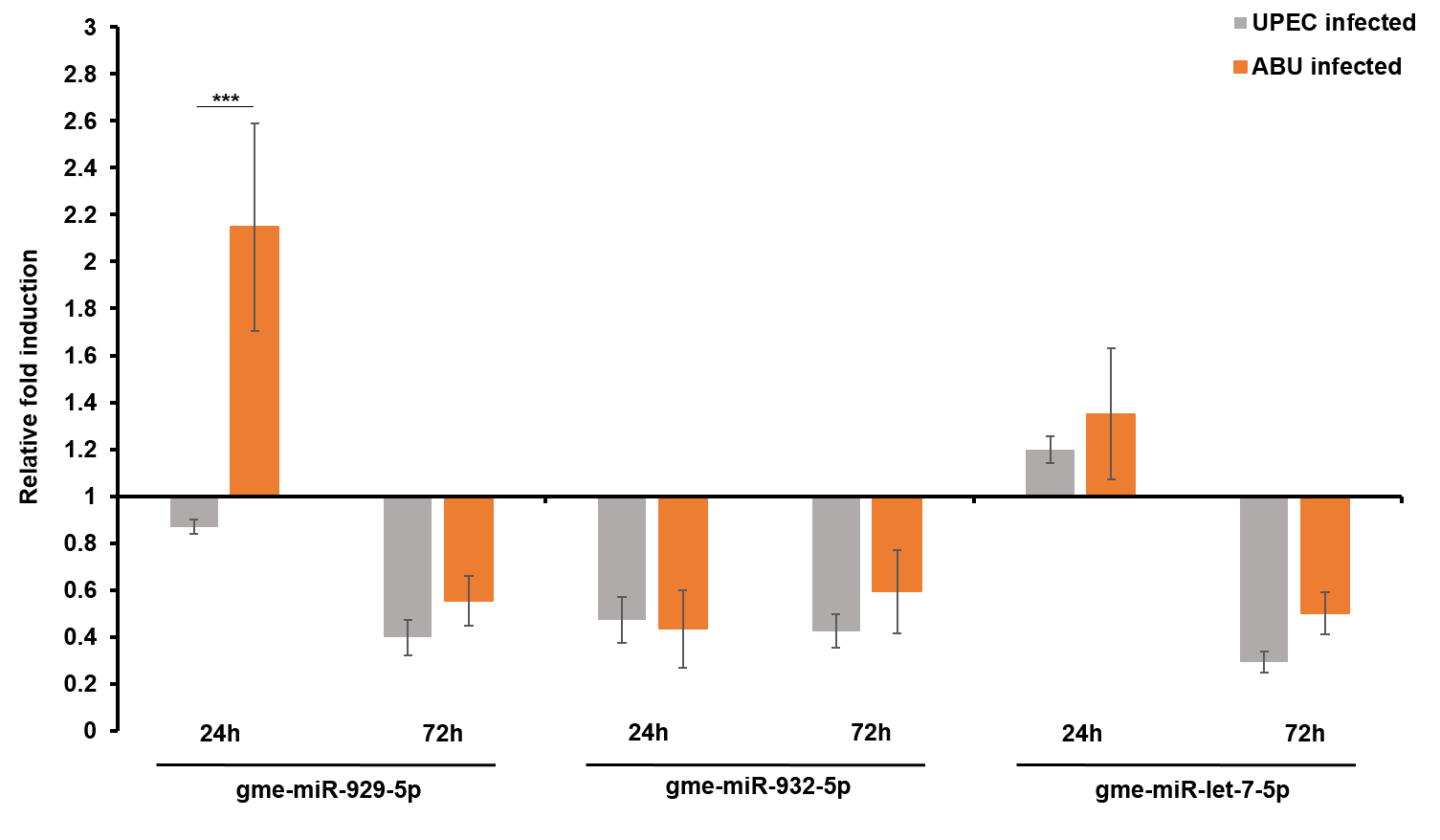


Supplementary Fig. S7. **Confirmation of the differential expression of selected miRNAs by RT‑PCR in *G. mellonella* larvae infected with ABU and UPEC strains, and in mock-injected controls**. The conserved miRNA sequences were obtained from small RNA sequencing by RT-PCR to confirm the differential expression of gme-miR-929-5p, gme-miR-932-5p, gme-miR-let-7-5p. The relative fold differences indicated for the miRNAs are normalized against mock-injected control and gme-miR-133 as the internal reference control (***p < 0.0001).

| **Supplementary Table S2. UPEC and ABU specific miRNAs** | |
| --- | --- |
| UPEC specific | ABU specific |
| gme-new-10-3p  (5’-CGTGGTCTCTTAGTGGCATC-3’)  gme-new-70-5p  (5’-GTAAGTAAAACACGTTGATTCA-3’)  gme-new-87-5p  (5’-AACAACAGGAATGTTATGTAC-3’)  gme-new-88-5p  (5’-TGTAGGGTTATTGTAGAATACA-3’)  gme-new-88-3p  (5’-TATTCTATAATTTCTTTGCTGCT-3’)  gme-new-89-5p  (5’-AGGCCATGCAGGCGAAGGAAT-3’)  gme-miR-92b-5p  (5’-AGGACGCGATTTGGTGTAAACCTTG-3’)  gme-new-117-5p  (5’- TTTTTCACCAGCGAAGTCAGA-3’)  gme-new-127-3p  (5’-TAATGCCTTCCTCTAACTACA-3’)  gme-new-145-3p  (5’-TTTTCCATGAAGTCGCCATCC-3’)  gme-new-148-3p  (5’-ATTTGGTTCTCTCTAATAGCAAT-3’)  gme-new-150-3p  (5’-CAATACTATGGTCAAGTGAGAA-3’)  gme-new-156-3p  (5’-TGAGGATGAAAACGGATCGATT-3’)  gme-new-157-3p  (5’-CAACGTTTAATACCACTTTGGA-3’)  gme-miR-274-3p  (5’-CTCGTTTTGACGATCGCAAAATG-3’)  gme-miR-307-5p  (5’-ACTCACTCAACCTGGGTGTG-3’)  gme-miR-929-3p  (5’-CTCCCTAATCGAGTCAGGTTGA-3’)  gme-miR-2756b-3p  (5’-CCCCTACGCTGCTACATTGTAT-3’) | gme-new-78-5p  (5’-TAGCATTACTACTTCACGAC-3’)  gme-new-135-5p  (5’-CTCATTATTTCGTCTGAAAAGAAA-3’)  gme-new-136-5p  (5’-TGTGAATTAAGAATACT-3’)  gme-new-137-5p  (5’-TGTATTTCAACACAGTAGAGATG-3’)  gme-new-161-3p  (5’-TACTTGTCTAGAGGGCGTGA-3’) |
|  | |
| **Supplementary Table S3. MiRNA and mRNA sequences** | |
| miRNA | Target mRNA |
| gme-new-70-3p  5’-GATCAATTTGTTTTCTTTACAGC-3’ | Gene 1  5’-ATGGGCTCCCTGCCTCGCATGTCGGTGGTGAGCGGCGGGCGGGCGGTGGTACCGGCTGCGCCGCTGCCGACCCACCTGGCACGGCTGGCCGCCAGCCAGGAGACGGCTCTTGTATATTCAGATGAAACCTGCAATGCTCGTATCAGTTATGTGGAAATGGAAGCTCAGACGAATGCGATAGCGAAAGCGTTGTCTAAACGCGCCAGACCGACTGGCGCCAACAGGGACGGTGACTATGTGATAGCTGTGTGCATGCAACCTACACACAATACAGTGTTAACATTATTAGCGACTTGGAAGACGGGGGCAGCGTATGTGCCGATGGAACCCAGCTTCCCACAAGCGAGGATATCACACATACTGCAAGACGCTGAGCCGGCCTTAGTTATTTATGATGATAGTGCAAATCCAGCTATGTTCGCCGGCAGTGGCATCCCATCGGTGTCTTTTGAAGAATTGATTCAAGAAGCCAGTGGACTATCCGCCGAGGAACTCAAAAGTCCGGAAGTGTTGGCTCACGCCGGAACAGACAGTATTGCTATCGTACTGTACACATCTGGAAGCACGGGTGTGCCGAAAGGTGTCCGTCTCCCTTATTCGGCTATATGCAACCGACTCTGGTGGCAATTCCGGACCTTCCCCTATTCCAATACAGAGAAGACCTGTGTTTGGAAGACGGCTTTGACCTTTGTGGATTCAGTCTGTGAAATTTGGGGCCCTCTTCTACACGGTAGGACTCTGTTAATCCTATCGAAAGAGACGACTAGGGATCCACAAAAATTGGTACGAGTTTTAGCTGAGAATCAGGTTGAAAGATTAGTACTAGTGCCGACTCTCCTTCGTTCTATCCTAATGTATCTATCCCTCACACCTTCCGAAAGGCCTTTACAGTACCTAAAGCTTTGGGTCTGCTCAGGAGAGACCCTAAGTAAAGAGTTAACAACCCAGTTCTTCCGATACTTCGGTGACAATGGTGGATACAAACTGGCGAACTTCTACGGCAGTACCGAAGTTATGGGAGACGTCACTTACTATGTACTGGAGAAACTTAATCAGTTAGACGTATATAATACTGTTCCTATCGGTTCCCCATTAGACAACTGTGCAGTGTACCTCTTAGACGAGGAGATGAATCCAGCCCGTGAGACTGAACCAGGGGAAGTGTGGGTCGCTGGACATAACCTAGCAGCTGGGTACGTGGGAGCTCAGGGTGCTGATAAATTCTGTGACAACCCGCATGCTGACCATCCAGACTTTAGCCGTCTATATCGCACTGGCGACTTTGGGATCCTACAGAAGGGAGTGATCCTTTATGCTGGACGTACCGACTCTCAAGTCAAAATTAGAGGTCACAGGGTCGATTTGCAAGAGGTTGATCGCGCCGTGACTGCAGTACCTGGCATTGAAAAATGCGTTGTACTATGCTACGGCTTGGAACGAGGGAACCCTGAAATCTTAGCATTTGTCACCATAGAACCAAGTGCACGCATCGCCGCGCATCATATTGAGGCCAGCTTGAAGAACTCTTTAACTAGCTACATGATACCGCAGGTAATTGTGATAGAAAGCATCCCCTTGCTTGTGAATGGGAAAGTGGACCGGCAAGCATTGCTGAAGATGTACGAGAATACAAACAATAATGACGACTCTGCAATCGCATTAGACTTTGACTATACAGGCGTAGATGAACAAGATAAAGAAGCGGCTAAAGTTCTCTTCGAAACAGTGGGAGAAGTGCTGGGGCGCGCCGCCAGAGGAACTTTGTCCGTAAGAGCCGGCTTCTACGAACTGGGAGGGAATTCTCTCAACTCCATCTACACGATCACCAAGTTGAGAGAAAAAGGATACTATATCGAAATCAGCGAGTTCCTGGGCGCAGCCAACCTTGGTGAGGTACTGGCCAACATGAGTACGAGCCCCGACAGTGGAGCGGACAGCAACGAGCCCAAGTTCGCAGCCGAGCTCATGAAGGATGAGGACAAACAGCAGGTTATCGACATGATAGTATCATCATTCTACGAGAAAGCGGAGTTGGAACAGTTCCTGAAGCACGAGATTGACACCATGGATTACGCACACTGCATAGACGCGTGCTGGGCTGCACTGCTACGAGCCAGGCTCAGCGTGGTGCTGAGAGATGGTACAAACACGCCGGTGGCAGTGGCTCTAAACTTCGACGCTCGGGACGAACCTGAAATTGAATTGACCGGTGGACTTGCCAAGATAATGGGATTTTTAGAGTTTGTTGAGGGCTCTGTGAGAGATACGTTATTACCGGAAGGCAAAGGCACGATTCTCCACTCGTTCATGATGGCCACGAACGCGGAACTGTCTCCGCGAGACAACGTCGCCGCGATACGGGCGTTAGAGCATGCCACCATGAGGATTGCGAGGGACAGGCGGTTCAAAGGCGTGTTCACCACAAACACTAGTCCACTTACACAGCAATTAGGAACCGATGTGCTCGGCTTCCAAACACTCCTCGACTATCAGATCAATCAATACGTAGATTCCAATGGAGACAGGACCTTCGGGAAAGCACCAGATGAC3’ |
| gme-new-40-3p  5’-CCTGCTGCTTAAGTGCTTATC-3’ | Gene 2  5’-ATGGTTCCTGCACCGAGCTGCCCGTCCACTTGGGACTACTGGACGACATCGCCGTCCGACTACGTGGAGCTGACATGCCTCCTGCCAAATACTATTTACATTCCTCTGAGAGTGAGTTGGGATGCTACTCTTCAGGATGTGAAAGAGGAGTTATGGGAGAAGGCGGCACATTATCCCTTATTTGGGGTGATGCATGAAATGTCAGGTTATGTGTTCCAATTTGTCAACTCCCTGGCAGTTCTTGAGGAGGTGGATGATGAGAACAAAAGGCTGCGAGATATCAAACCAGTATGTGGAGTGCTTATGATCATAAAGAGGTCTGTGAAACCAGGAGAATATCTGCTGAACACACAAATAAGCCATTTAATTGGAAAAGGTTTAAACGAATTTGATAGTCTAAGAAGTAATGAAGTGAATGATTTTCGGACACAAATGCACAATTTAGCTGAAGAGAGTTTATTAAGACGAATGAAAAGTGATTGGCAAGAGAAACTGCGATATCACTACCCACCAAGACTGGCTGATCAACCTATACCTACCACACTCAAGAACCAACTCAATAGAAATAGTTTTATGCTTGTCACTAGATTTGCTAATTCTGAGTTTTCATCATCTCTTTGCGTACCATTCACACAAACGCCGCAGCAGCACATCGAGATCATTCTCAGAAAGCAAGCGAAGTCACTGAACATACGCGGCGAGCACCCACACAATTATGTTTTGAAAGTGTGCGGCCGCGAGGAGTATTTATTCGGGGATTACCCGCTCATACAGTTCAAATACGTCCAGGAGATGCTGTCTCGGGACTCCGTGCCCCAAGTCATGACTGTCAGTGTGGACAAATTGAGATTTTTAAATGCCGACCCCCAGCAGTACTATATACGTGAACAGAGGCGGCAGACCGCTGACTCGAATACAATGAAAAGACGGAAGAATGAACTCTCCTGGGATATAGACAAGTTATACTCTTGCATGGTGCTGAGTGTTGGCGGGTTGAACGTGGACCCTAATCGTGTTGTTGAGGTAATTTGTCAAGCTGGCGTATTTCACGGCGGTAAGCCTTTATGCGAGGCGCAGAAGACTCGAGCGGCGGCGGTGTCGTCGGAGGGCGTTGCGCAGTGGCAGCAAGAACTCAAGTTCCCGCTCAAGGTCTACAACATACCGAGAATGGCGAGACTATGCTTTGGGATATATGAAATTGAAATTAATAAGACTAAGAATAAGAAGAAGGGGAAGGACTCCGGCAAGGACTCAATAAACCGGCTGGCGTGGGCGAACACAATGATATTCGACTACAAGGATCAACTGAGGACGGATAAAGTGTCATTTTTCATGTCAACTCACGTGGCGGATGAAACGCAGGGCGACGATCAGCTGCTGCATCCCCTGGGGACAGTGTTCTCCAATTCGAATACAGACTCGTGTAGCGCCGTTTTACATGTGCAGTTCTCAAATTATGATTGCCAATATCCTATTGTATTTCCGAAACAAGAAATGGTAAAAGCTTATGCGGAGCGCATTGAGAATGGGTCACCCGAAGTGATGTCGCGCCTTAAGAGAGATTTCGAGAAGCTTCGAGCGACCGCCGAAAAGGATCCCATGTATGAAATGCATGAGCAAGACAAGAAGAATATATGGGCGTTGAGAAACGACTTCCGCTCACTGGCCCCGTGGTTGCTGCCGCGCCTGCTGTGCTGCGTGGAGTGGGGCGAGCGGGCGGAGGCGGCGGCGGTGGCGCGGCTGCTGGACGACTGGCCCATCTCGCTGCCGGTCGAGTCCGCGCTCGAGTTACTCGACTACGCGTACGCGGACGCCACCGTCCGGAGCTTCGCTGTTCGGTGTCTGCAGAAGATCAGTGACGAAGACCTCCTGTTATATCTATTACAACTGGTTCAAGCGTTGAAACACGAGCCCTACCTCATGTGTGATTTGTCGGTGTTTTTGTTACAACGCGCGTTTAAAAACATGATCATTGGTCACTATCTTTTCTGGCATTTAAGATCGGAGATGCACATGCCGTCGGTGTCGGTGCGGTTTGGTCTACTGTTGGAGGCGTACTGCCGCGGCTGCCAAGACCACATCAGTATTCTGCTGCGGCAAATCGCATGTCTCGACAAACTCAAGTGGGTGAGCCAGAATGTCCGCAAGAAGAAGGAAATATCAAAAGCGCGGGCAGCGCTGCAGCAGAATTTGCAACAGACGCATTGCATCGAAACGCTCTGCGACTTCGTATCGCCACTTAATCCGAGCTACCGCTGCAAACGGATACAGCCGGAGAAATGCCGCGTTATGGACAGCAAGATGCGCCCTCTGATGGTGGACTTCGAGAATAGTGACCCGTTCGGCTCAGATATCCGGATCATACTGAAGATCGGCGACGACCTTCGTCAGGATATGTTCACATTGCAAATGCTCAGGATAATGGACAGGCTGTGGAAGAGTCACGGTTATGACTTTAGGTTAAGTCCATACAATTGTATTTCAATGGAGAACGAAGTGGGTATGATCGAGGTGGTGGAGGACGCGGAAACGGTTGCTAATATACAAAAACAACCCGCCATGTTCCAAGCCGCCTCCACAATGTACAAAGGGACTTTGCTACAATGGCTAAAGAAGCAGACAGAGGACGAGTGCGGGCGTCCCAACGAGGCGGCGTTCAACAAAGCAGTGGACGAGTTCACAATGAGCTGCGCCGGCTACTGTGTCGCCACCTACGTCCTCGGTATCGCGGACCGACACCCGGACAACATTATGGTCAAGAAAAGTGGACAGCTATTCCACATAGACTTCGGCCATTTCCTCGGCCACTTCAAACAGAAGTATGGGTTTAAGCGCGAACGTGTGCCGTTCGTTTTAACGCACGATTTTATACACGTGATCAACAAGGGGCAGCGGGGCTCGGGCGATAATGAGCCCATCGACTTCAAGATATTCAAAGAGCACTGTGATACGGCATTCAGAATACTCCGAAAGCATGGCCACCTCATCTTGTCTCTCTTCTCGATGATGATCTCTACCGGCCTTCGGGAGCTGAGCTCCGAGAAGGACTTGCAGTACCTTAGAGAAACCCTCGTAATGGATTTATCCGAGGAGAAAGCCATGGAGCACTTCAGGTCGAAGTTCAGCGAGGCGATGAAGAACTCATGGACGACATCGCTCAACTGGGCGTTCCATAATATCGACAAAAACAACTGA-3’ |
| gme-new-138-3p  5’-ATATTATGCACATTACTATGGAT-3’ | Gene 3  5’-ATGAAATTTACAATTGTCATAAATATTACAAATAAGGATGCAGCAACTACATCTGAGTTATTTAATAAAAAACCAGATAAGTCCGTAACCATTTTTGATTTGGTTATACACTTTTATAATTTTGACTATCAGAATAGTACTACACATGTACCTGAACACTCATACATAGCAATGACATCTGGCAGCACTGGAGAACCAAAGCACATACAAGTGCCTGTGCAATGTATTCAACCAAATATAGATGATTTAACTAAATTATTTAATATTACTGCTGATGATATAATATATTTCTCTACACCACTAACATTTGACCCATCCATGATAGAGATACTGCTCGCCTGTATGAATGGAGCCTCTCTACTTATTGCACCTGAAAAAGCAGACATATTATTCCCCAACAACAAAGAGAATTCTGTGACATTTTGGCAACTTACACCATCACGATTTTTTCAGCATTCAAATTCTGATATCAAGAATAAAATATTAAGTGCAAATTCAACATTGAAAATACTAGCTTTAGGTGGTGAGCCATTAAATGGTGTGAAGAGACTAAAGGAATTGAAAGATTGGGATAATAAAACTAGAATATTCACATTGTATGGAGTAACTGAAATGTCATGCTGGGCTTGTGTTGCTGAGTTAGATCTTAACAAAATACTAACTGACAAAGAAATACCATTAGGTAACTGTCTGTCAGAAACAGAATTACATGTGGAATCAAATGATGACAATAAAGAATGTGGAAAAATTATTTTAGTAAGCAAAACAAGAAAGTGTGTTATATTAAACAAAACCATTGGAAATGAAGATGAAAATTCTTTAAAATTTATTGACACTGGAGATTTAGGTGAAGTAAGAAATGGCACTGTGTATTATCGGGGCCGCAAAGATGATATTATTAAAAGATTTGGACACAAAATTAATTTACAGTTTATTGAATCAACTATAATGCAATGTCCGAGTGTGAAAACAAGCTCCTGTATCTGGCTCCCAAAATCATTACTTCTGATTGCCTATTTCTCATCAGAAACACTTAGCAGCCAAGAGTTGTCCAACTTTTTGAAATGTAAACTTGATGATAAGCACTGGCCAGATAAAATAATTAGAGTTGACAATTTACCAACAAATCCTCATGGGAAGATATCTAAATTGATATTATCTAAAATGTATGAAAAAACAATGAACACACCACAGACATTAGATTCCTTAAAAGTGAGTTTCTTAAAGGAACTTCAAGCTGTAATGGGTCAACACTTCACTTATGATCAAATTAAAAGTAAAGACTTCTTTGCCATTGGTGGCACATCTTTTCTAGCGATATCTATGTGTAATAAGCTTTCACTACTTTGTCCAAAATTTGGTAAACTAATTCTCCCTTACTTGATGTCTCAAAAAAATACTATAGATGATATTATGCAATTGGCATATAAAGAAATACATGTTGATGAAATAAAAGTTAAGAAAAAAATTAAAAGGTCACGGTCAGATGCAGGTGGTTATGTAGAGAGTCAGTCTTACAAAAGAACTAACACAAAAAGTCTGACAAATCCTGTAAAATTCATTGTGTTATGGTCATATGATACTGGAAAATGTGTAGATGCTTCTCCATCTTTATTTCAAATAGGATTCAATTTATATGTGACAGTCGGGAGTCATTCAGGGAAGATTATAGTCATGGATGCGATATCTGGAATATTGCAGGGAATGGTAACAGTAAAATCACGTGTTGAAGCATCTGTATTTTGTTACCACAAGAGAGACATGTCGCCGTGCGGTGTGGTTGGCACTTACGATGGCACAGTGGTATGCTTCCAATTAGAAACATGCAAAGAGTTGTGGAGAATCAACATTGGATCAATGATAAAAAGTAAAGCAACATGTTGCAATGATCTACTCTACATTGCTTCCTATGATGGAAAGATAAGATGTATAGATATTGCGATAGGGGTAATCAAAGAGACTATATATGTGGCAGATCAAGGTATATCAGCTGATCTAGTACTTGCTAAAAACAAGTATGTGTTAACCAGTACGCTGTCTGGTGTGTGTGCAAGTATACATACTCTAACCAATACTGTGGCTTGGCGCTGCACACTGAGTAGTCCAGTATTTGCAAGTCCTGTGCTTTACGATGACGACAAGTATGTGGTATTCGCGGAAGTTAATGGTGAAATACATTGCAGGACCGTTGAAAAGGGTATTAAGATATGGAATTATCAAGGAGCAAGAGGTAACATTTTTTCCTCCCTCTACATAAAAGAAGTTGATAAACTGAAATGGCAAATGGTTTTTGGCTGTCACGATAACAAAGTTTACAGCATTAATATCAAGAATTTCCAACCTAGCTTGAATTGGAAAGCACAACTCACATCACCTGTATACTCCACTCCATGTGGTCTAAGTGACAAATTAATACTTGCTGCCTCTAATAATGGCAGGTTATGCGTTATAGATGAAGAAAACGGGATAATATTGGCAGAACATCATCTGCCAAATGAAACATTTTCATCACCAGCAGTTTATGGAGATTACATATTCATTGGTTGCAGGAATGACCACCTTTACTCTTTGAAATATATTTTAAATTTATAA-3’ |
| gme-new-4-5p  5’-TGGTTTTCATAATGATTTGACAGA-3’ | Gene 4  5’-TTGAAGCCTACAGAAATGTTTATAGACGAAAATGAGACGCCGGAGACATGGTGGGTGTCGGCGGTGCTCAAGACCATCAAGGCGGTGACGCTGGTCTTCGACATCCTCACCTTCCCCATTCACTTAATAGTGCAGCGACCATGGAGGAAGCGCGCCCTCTCGCGCCGGATTAAGGCGCGCATCACGCAGTCGTCGCCGGGCTGCGTGACGGTGCGGTCGGTGTCGTCGCCGGGCGAGCTGCACGTGCGGCTGGTGCGCGACGGCGTGCTCACCATGGAGAGCATGCTGCGCGCCGCCGCCCAGCGCTGGGGCTCGCGCCGCTGCCTCGGCACGCGCACCGTGCTCAGCGAGGAGGACGAGCCGCAGCCCAACGGCAGGCTCTTCAAGAAGTTCAAAATGGGCGATTACGTGTGGCGCACGTACACGGAGGTGGAGGCGGAGGCGCGGCAGTTCGCGAGCGGGCTACGCGCGCTGGGCTGCGCGCCGCGCGCCAACATCGCCATGTTCGCCGAGACGCGCGCCGAGTGGATGCTCGCCGCGCACGGCTGCTTCAAGCTCAGCATCCCAGTGGTAACAATCTACGCGACGCTCGGCGACGAGGCCATAGCACACGGCATCAACGAGACGGAGGTGTCCACTGTCATCACCACTCACGACCTGCTGCCCAAGTTCAAGAAGATCCTCGCCAAGACGCCCAAAGTGGACACCATCATATACATGGAGGACCAGCTGCAGACCATCGACCGGGAGGGCTACAAGCCCGGCATCAGGATCGTTGGCTACAAGGAGGTCATACAGAAAGGAATAAACGCCAGCTTTGAGGCGGTGCCGCCGGCGCCGACGGACACGGCCATCATCATGTACACGTCGGGCTCGACGGGCGTGCCCAAGGGCGTCATCCTGTCGCACCGCAACATGCTGGCCACGCTCAAGGCGTTCGCGGACGTCGTGCCCATATACGAGGACGACATGCTCATGGGCTTCCTGCCGCTCGCGCACGTCTTCGAACTATTGGCCGAGAGTCTCTGCATCATTGGTGGTGTCCCCATCGGGTACTCGACGCCGCTGACGATGCTGGACTCCTCCAGCAAGATCATGAAGGGCACCAGCGGAGATGCCACCGTCCTCAAGCCCACCTGCATGACCACAGTGCCGTTGATAATGGACCGCATCAGCAAGGGCATCACGGACAAGGTGTCTCGCAGCGGGCCGTTCGCGAGCGCGTTCTTCCGCTGGGCGTACTCGTACAAGCAGACGTGGATGCGGCGCGGATACGACACGCCCATACTTAACAGGATTATGTTCAGCAAGATCCTGGGCCTGCTGGGCGGGCGGCTGCGGCTGCTGCTGGCGGGCGGCGCGCCGCTGGCGCCGGACACGCACCAGCAGCTGCGCATCTGCCTGTGCTGCGACGTGGTGGCGGGCTACGGGCTCACCGAGACCACGTCGGCCGCCACCGTCATGGACGCGCACGACCGCTCCACCGGCCGCGTGGGCGCGCCCTCGCCCGGCACCGCGCTGCGCCTGCTCGACTGGGCCGAGGGCGGCTACCGCGTCGCCAACAGGCCCTTCCCGCAGGGCGAGATCGTGATCGGAGGTGATTGCGTAGCGGAGGGCTACTACAAGAATCCAGAGAAGACCCGGGAGGAATTCATCGAGGAGGACGGCATTCGCTGGTTCAGGTCTGGGGACATCGGCGAACTGCATCACGACGGCTGCATCAAGATCATCGACCGCAAGAAGGACCTAGTGAAGCTGCAGGCCGGCGAGTACGTGTCCCTGGGCAAGGTGGAGGCGGAGCTGAAGACGTGCCCCATCGTGGAGAACATCTGCGTGTACGGCGACAGCTCCAAGACGTACACCGTGGCGCTGGTGGTGCCCAACCCGCGGCACCTGGCCGAGCTGGCCGCGCGGCTCGGCCTGCCCGACCGGGACTTCGACCAGCTCTGCCACAACACCGCCGTCGAGAAGGCCGTCGTCAAGGAGCTCGCCGACCACGCTAGGAAGTGTGGGCTGGAGAAATTCGAGGTTCCTGCTGCAGTGAAGCTGTGCACGGAGGTGTGGTCCCCCGACATGGGGCTAGTTACCGCCGCCTTCAAGATCAAGCGGAAAGACATCCAGGAGCGGTACAAGGAAGACATCAAACGAATGTACGCCTCCTGA-3’ |
| gme-new-135-5p  5’-CTCATTATTTCGTCTGAAAAGAAA-3’ | Gene 5  5’-CCATGTTCATTGCCAGTCGTTTGTGGTTGCGCGCGAACATACGTCGTCCATTTTATATTTAAGTTTTTTTTTTATATATTTTTTTTAATTTCCGCCACGATGGCGTCAGCCGTGATTAAGTTTAGTGCCATAATGATGCTAATCGGCGTGTGTGCAGCTGATGTCTCTGAACTCCCCGAAGTCAAGGCAGCAGCGGCCGCAGCCCCGCCCGTGACTGAGGTTTGTCTTGGCTGCATCTGCCAAGCCGTGTCGGGGTGCAAGCAAGGAACTCAATGCGAAGGAGACCATTGTGGTCTATTCCACATCACTTGGCCATACTGGGCTGATGCCGGGAAACCAACGATTAATGGACTCTCACCTGACGATCCTAATGCGTACCCCAGTTGCACCAATGACCCGTACTGCGCTGCGCAGACCGTACAAGGCTACATGAAGAGATACGCTCAGGACTGCAACGGCGATGGTCAGATAAACTGCTACGATTACATGGCCATCCACAAGAAGGGAGGGTACGGGTGCAGTGGGGAGCTGCCCTTCAATTACGTCAACACTTTCAACCAGTGTGTGGCAATTGTTGCTTCTCAG-3’ |
| gme-new-121-3p  5’-TATTGCTTGAGAATACACGTAG-3’ | Gene 6  5’-ATGAGTGACATGTTGAATTATGGTTGCACTAGGCTTGAAATTGGTGTTCAATCGGTTTATGAGGACATTGCTCGTGATACTAATAGGGGACACACAGTAAAAGCTGTCTGTGAGAATTTTAATTTGGCCAAGGATGCTGGATATAAGATTGTTGCGCATATGATGCCTGATTTACCCAATGTGGATTTTGAACGTGATGTGGAACAATTTATTGAATTCTTTGAAAATCCCGCATTTCGAGCTGACGGCCTTAAGATATACCCGACTTTAGTTATTAGAGGTACTGGTCTATATGAACTATGGAAGACTGGGCGATACAGAAGTTATCCTCCATCAACTTTGGTTGATTTGATTGCAAAAATACTGGCATTGGTACCACCATGGACTAGGGTCTACAGGGTCCAACGTGACATTCCCATGCCCCTTGTTTCATCCGGAGTGGAACATGGCAACTTGAGGGAGTTGGCGCTAGCTCGCATGGCTGACTTGGGTACAGATTGCAGAGACGTGAGGACCAGGGAAGTAGGGATACAGGAGATACATAACAGAGTTAGGCCATACGAGGTAGAGTTAATAAGACGAGATTATGTTGCCAATGGTGGATGGGAGACATTCCTAGCATACGAGGATCCAGATCAGGATATATTGGTAGGCCTCCTGAGGCTCAGGAAATGTGCCTCGGACACTTACCGGCCAGAATTGAAACCTGGTCCAAATTCAAATTTCAAGCAATGTAGTATAGTCAGAGAACTGCATGTTTATGGATCAGTTGTACCTGTGAATGCCCGCGACCCAACAAAATTCCAACACCAAGGCTTTGGGATGTTGCTAATGGAAGAAGCGGAGAGGATAGCTAAAGAGGAA-3’ |
| gme-new-70-3p  5’- GATCAATTTGTTTTCTTTACAGC-3’ | Gene 7  5’- ATGAGTGACATGTTGAATTATGGTTGCACTAGGCTTGAAATTGGTGTTCAATCGGTTTATGAGGACATTGCTCGTGATACTAATAGGGGACACACAGTAAAAGCTGTCTGTGAGAATTTTAATTTGGCCAAGGATGCTGGATATAAGATTGTTGCGCATATGATGCCTGATTTACCCAATGTGGATTTTGAACGTGATGTGGAACAATTTATTGAATTCTTTGAAAATCCCGCATTTCGAGCTGACGGCCTTAAGATATACCCGACTTTAGTTATTAGAGGTACTGGTCTATATGAACTATGGAAGACTGGGCGATACAGAAGTTATCCTCCATCAACTTTGGTTGATTTGATTGCAAAAATACTGGCATTGGTACCACCATGGACTAGGGTCTACAGGGTCCAACGTGACATTCCCATGCCCCTTGTTTCATCCGGAGTGGAACATGGCAACTTGAGGGAGTTGGCGCTAGCTCGCATGGCTGACTTGGGTACAGATTGCAGAGACGTGAGGACCAGGGAAGTAGGGATACAGGAGATACATAACAGAGTTAGGCCATACGAGGTAGAGTTAATAAGACGAGATTATGTTGCCAATGGTGGATGGGAGACATTCCTAGCATACGAGGATCCAGATCAGGATATATTGGTAGGCCTCCTGAGGCTCAGGAAATGTGCCTCGGACACTTACCGGCCAGAATTGAAACCTGGTCCAAATTCAAATTTCAAGCAATGTAGTATAGTCAGAGAACTGCATGTTTATGGATCAGTTGTACCTGTGAATGCCCGCGACCCAACAAAATTCCAACACCAAGGCTTTGGGATGTTGCTAATGGAAGAAGCGGAGAGGATAGCTAAAGAGGAA-3’ |
| gme-new-147-3p  5’- ATTTGGTTCTCTCTAATAGCAAT-3’ | Gene 8  5’- ATGGCTATGCAACCACACAGTAAGAAAAGAGTCTGCTACTATTATGATAGTGATATTGGGAATTACTATTATGGACAAGGTCATCCCATGAAACCTCATCGCATACGCATGACACATAATTTACTTCTAAATTACGGCTTGTACAGAAAAATGGAAATTTATAGACCACATAAGGCGACAGCTGATGAGATGACAAAGTTTCATTCGGATGACTACATTCGCTTCCTGCGCTCCATCAGACCGGATAATATGTCTGAATATAACAAACAAATGCAGAGATTCAATGTTGGTGAAGACTGTCCAGTGTTTGACGGCTTGTATGAGTTTTGTCAATTGTCTGCTGGAGGTTCTGTTGCCGCTGCTGTTAAATTAAATAAACAGGCATCAGAAATCTGCATCAACTGGGGTGGTGGCCTTCACCACGCAAAGAAGTCGGAAGCATCAGGTTTCTGCTATGTAAATGATATTGTACTTGGCATATTGGAGTTACTGAAGTATCATCAAAGAGTACTGTATATTGATATTGACGTACACCACGGTGATGGGGTCGAGGAAGCTTTTTACACCACAGACAGAGTAATGACTGTCTCCTTCCATAAGTATGGGGAATACTTCCCTGGAACAGGTGATCTCCGGGATATCGGTGCCGGCAAGGGCAAATACTATGCTGTGAATATTCCCTTGCGTGACGGTATGGACGATGAATCATACGAGTCAATTTTCGTACCCATCATATCCAAAGTCATGGAGACCTTCCAGCCGAGTGCGGTGGTACTTCAATGTGGAGCTGATTCACTTACTGGTGATAGATTGGGCTGTTTTAATTTAACAGTCAGAGGTCACGGCCGATGTGTGGAGTTAGTAAAGCGATTTGGCCTACCTTTCCTTCTTGTGGGAGGTGGAGGATATACGATCCGCAACGTGTCACGGTGCTGGACATACGAAACATCAGTAGCACTAGGCGTTGAGATCGCTAACGAGCTGCCCTACAATGACTACTTTGAATACTTCGGTCCGGACTTCAAACTGCACATATCACCCAGCAATATGTCCAATCAGAACACTCCAGAGTACTTGGAGAAGATTAAAAATAGGCTCTTTGAGAATCTACGGATGTTGCCGCATGCACCTGGTGTACAGGTACAAGCCATTCCAGAAGATGCGGTAAATGATGAGTCAGAAGACGAAGATAAGATTGACAAAGATGAAAGGCTGCCACAAAGTGAAAAGGATAAACGCATTACGGGTGACGGCGAGTTGTCAGACTCTGAAGACGAGGGTGAAGGCGGGCGGCGCGACAACCGCTCGTACCGCGCGCCGCAACGTAAGAGGCCCCGTCTCGACAAGGACGGCTCGCAGATCAAAGACGAAATTAAAACTGAAGATATAAAAGATGACGTGAAGAATGTGAGCAGTGTAGAGGAACCAAAAAAGGAAGTGCCACCCAATCCCTGA-3’ |
| gme-new-160-5p  5’- GTCATTCAGCCTGCCAGCATTGCT-3’ | Gene 9  5’- ATGCCAAATATTTTGTTAAACTTTGTTGTTCTTTCATTCGCTGATCTCAAGAAATTTCATTACTACTACTGGTTTGCATTTCCCACTCCTAGTCAGCCCACAGTCTATTTAAATGAAAAAGCAAAATTGATAACAACTCAGTTCAGTTTCGATCAACTTCAATTATTAGTCCAAGGGTACAATTCATTAGATTCAAGTCAAAAATCATTCTTCATTGTTACCAAAAGTGATGATAAGTTGTCAGTAATTCCATTGTCAAATATTTTACAACCAAACTGCAATGAATTAAGTCTTGATTTATCAGATGTGTATTTTGTGTTTGCTGATCCAAGTAACACCGATAATCCTGGGTGGCCTTTAAGAATTTTCTTAGCAGCTTTACTTGATCATTGCACAAATCTTTCTGGGAAAAATATTCAAGTCATTGGTTTGAGGTGCAATGTTAAATATGAAATTGCAAACAGTCTTGTTTATTCTATCTACATTTCTCAGGATATTCAATCAGCAGAGAATGCAGGTTGGGTTGGATGGGAGAGAAATGATAAAGGCAATTTTGGCCCTAAACTGGCTAATATGTCAGCGTCAATGGATCCTGTAGTTTTAGCTGATACGTCATCAGATTTGAACATCAAACTAATGAAATGGCGTTTAGTACCTAATATAGATGTAGAGGTGATGAAGAGTACTAAATGTTTATTATTGGGTGCTGGTACGCTTGGATGTCATGTGGCTCGTAATCTGTTGGCTTGGGGATTTCGTCATATAACATTTATTGACAACGGTAAAGTGTCGTACTCAAATCCGACGCGACAAGTACTCTTCAATTATCAAGATTGTCTAAATGGAGGACGTAAGAAAGCTGAAGCAGCGGCCGACAATCTTAAAAATATACTACCTACTGTAACTGCCAAGGGTTTAGTAGCTCATATACCAATGCCTGGTCATCCTATTGGAGAATCTCTTAAGGCCGAAACTATATCCAATATCAAGACTATTACTGAAGCTATCGCAGATCACGATGTGATATTTTTGTTACTCGATACTAGAGAGGCAAGATGGTTGCCTACACTCGTCGCAGCACATTATGGAAAGATAGTAATCAATGCAGCGTTGGGTTTCGATAGTTACTTAGTGATGAGACATGGAATAGGTGGCACGCCATCAGAGGGCGCGACACTCGTCAATGCCACACACATAGCAGGTGGACAACTGGGTTGCTATTTCTGTAATGACGTCACTGCTCCAGGGAATTCCCTTAAAGATCGCACTCTTGACCAACAATGCACCGTAACACGGCCAGGCGTGGCGGCAATAGCCGGGGCGCTTGCTGTGGAAATTCTAGTTGGATTGTTGCAGCATCCTCTAAGGGTAGAAGCACCAGCGATATATAACTTGAATCAAGAAATTGACACCATATCATCAGATATGCAAGGTGTTTTGGGCCCCGTACCACACTCTATCAGAGGATTTCTCCATTCATACCAAACAGTAGTGCCAACATGCGGGAAATTCAAACAGTGCATAGCCTGTTCTGATAATGTTTTAAACAAGTACAAAGAAGCGGGAATGGAGTTCTTGTTCAATGTATTCAATAGTGGAAAATATTTGGAAGAAGTAACTGGATTAACAGAATTACAATTGTCTGCAGAAATGACCGATATATTGACATTTTCTGATGACGACGATAATGAATAA-3’ |
| gme-new-106-5p  5’- CCTTGTCATTCTTCTTGCCCAGT-3’ | Gene 10  5’- ATGATGTCGACTTCAACAGATACTTTATCACAAAACTTAAAAAGTGATGAAAATGGTGAATCTAAATCAAATGACACTGAACACCTTGAAGGAAATAAACTCTTGGTTAAAAGAAGAAATTGCCAAGACGAATTGGCGCCACCTTCAAAGTCTTTTAAAAATGATGAACATAGTACCGTAGTAGCAGCTCATTACAATCATCTTGAAGAAAAAGGACTAAAGGAAAGGTTCAAATCTCCCATATTTTACGTACGAAATTTTAATAACTGGGTAAAAAGTGTGCTCATTCAGGAGTACACAGATAAAGTGAGAGAAAAAGACTATGGTAAACCTATCATGGTGCTTGATATATGTTGCGGTAAAGGAGGAGACCTCAGCAAGTGGCAAAAAGCGCGTGTTGAAAAAGTGATATTTGCTGATATAGCGGATGTGTCTGTTCAGCAATGTAAAATTCGCTATGATGATTTACATAAAAGATGTGGCAGACTTTACTCTGCTGAATTCATTGCAGCTGATTGTACAAAAGAGACTCTGAGAGATAAATACTCAGACCCATCAATAAATTTTGATCTTGTAAGCTGTCAGTTTGGACTACATTATAGTTTTGAAAGTCTAGGCCAAGCTAGAAGAATGCTCACTAACATAACAGAGTGTCTCCGTCCAGGTGGATATTTTTTTGGTACTATTCCAGATGCATATGAAATTATTTCCCGAACTAAAAAGTCAGCTGATGGGTCTTTTGGAAATAGAATCTACAATATTAAGTTATTATTTGATTCCAAAACAGGTTATCCATTGTTTGGTGCAAAATATGATTTCCATTTAGAGGGAGTAGTAGATTGTCCTGAGTTCTTAGTTAATTTCGAACTATTTGTTAAACTAGCTGCTGAGTATGGACTTGAATTAGTATACAAAGCTAGGTTTTCAGATTTCTTCAAAGATCATTCAGATAACTATAAACAGTTACTGCATAGAATCATTTGTTTTGAAAGTTATCCAGCACCGCCTGGTAAAGAACTCATTGGAGATGAGGCAGAATATGAGCATGCAAAACAATTTTGGGAAAATATGGAGAAGAAAAATGAACATGATCATATTGGAACAATGAGTATGTGTGAATGGGAAGTAGCCACTATCTATATGGCATTTGCATTTAAGAAACAGAAGTCCACTTGGGATTCAAATGGAAAACCAGTATACAAATTGCCTCAAGATGAGGAGAAAGCAGAGTGA-3’ |
| gme-new-147-3p  5’- ATTTGGTTCTCTCTAATAGCAAT-3’ | Gene 11  5’- ATGGCTATGCAACCACACAGTAAGAAAAGAGTCTGCTACTATTATGATAGTGATATTGGGAATTACTATTATGGACAAGGTCATCCCATGAAACCTCATCGCATACGCATGACACATAATTTACTTCTAAATTACGGCTTGTACAGAAAAATGGAAATTTATAGACCACATAAGGCGACAGCTGATGAGATGACAAAGTTTCATTCGGATGACTACATTCGCTTCCTGCGCTCCATCAGACCGGATAATATGTCTGAATATAACAAACAAATGCAGAGATTCAATGTTGGTGAAGACTGTCCAGTGTTTGACGGCTTGTATGAGTTTTGTCAATTGTCTGCTGGAGGTTCTGTTGCCGCTGCTGTTAAATTAAATAAACAGGCATCAGAAATCTGCATCAACTGGGGTGGTGGCCTTCACCACGCAAAGAAGTCGGAAGCATCAGGTTTCTGCTATGTAAATGATATTGTACTTGGCATATTGGAGTTACTGAAGTATCATCAAAGAGTACTGTATATTGATATTGACGTACACCACGGTGATGGGGTCGAGGAAGCTTTTTACACCACAGACAGAGTAATGACTGTCTCCTTCCATAAGTATGGGGAATACTTCCCTGGAACAGGTGATCTCCGGGATATCGGTGCCGGCAAGGGCAAATACTATGCTGTGAATATTCCCTTGCGTGACGGTATGGACGATGAATCATACGAGTCAATTTTCGTACCCATCATATCCAAAGTCATGGAGACCTTCCAGCCGAGTGCGGTGGTACTTCAATGTGGAGCTGATTCACTTACTGGTGATAGATTGGGCTGTTTTAATTTAACAGTCAGAGGTCACGGCCGATGTGTGGAGTTAGTAAAGCGATTTGGCCTACCTTTCCTTCTTGTGGGAGGTGGAGGATATACGATCCGCAACGTGTCACGGTGCTGGACATACGAAACATCAGTAGCACTAGGCGTTGAGATCGCTAACGAGCTGCCCTACAATGACTACTTTGAATACTTCGGTCCGGACTTCAAACTGCACATATCACCCAGCAATATGTCCAATCAGAACACTCCAGAGTACTTGGAGAAGATTAAAAATAGGCTCTTTGAGAATCTACGGATGTTGCCGCATGCACCTGGTGTACAGGTACAAGCCATTCCAGAAGATGCGGTAAATGATGAGTCAGAAGACGAAGATAAGATTGACAAAGATGAAAGGCTGCCACAAAGTGAAAAGGATAAACGCATTACGGGTGACGGCGAGTTGTCAGACTCTGAAGACGAGGGTGAAGGCGGGCGGCGCGACAACCGCTCGTACCGCGCGCCGCAACGTAAGAGGCCCCGTCTCGACAAGGACGGCTCGCAGATCAAAGACGAAATTAAAACTGAAGATATAAAAGATGACGTGAAGAATGTGAGCAGTGTAGAGGAACCAAAAAAGGAAGTGCCACCCAATCCCTGA-3’ |
| gme-new-135-3p  5’- AAGTTTTCCGTGACGATATAAGGGGGCTCC-3’ | Gene 12  5’- TTGAAGCCTACAGAAATGTTTATAGACGAAAATGAGACGCCGGAGACATGGTGGGTGTCGGCGGTGCTCAAGACCATCAAGGCGGTGACGCTGGTCTTCGACATCCTCACCTTCCCCATTCACTTAATAGTGCAGCGACCATGGAGGAAGCGCGCCCTCTCGCGCCGGATTAAGGCGCGCATCACGCAGTCGTCGCCGGGCTGCGTGACGGTGCGGTCGGTGTCGTCGCCGGGCGAGCTGCACGTGCGGCTGGTGCGCGACGGCGTGCTCACCATGGAGAGCATGCTGCGCGCCGCCGCCCAGCGCTGGGGCTCGCGCCGCTGCCTCGGCACGCGCACCGTGCTCAGCGAGGAGGACGAGCCGCAGCCCAACGGCAGGCTCTTCAAGAAGTTCAAAATGGGCGATTACGTGTGGCGCACGTACACGGAGGTGGAGGCGGAGGCGCGGCAGTTCGCGAGCGGGCTACGCGCGCTGGGCTGCGCGCCGCGCGCCAACATCGCCATGTTCGCCGAGACGCGCGCCGAGTGGATGCTCGCCGCGCACGGCTGCTTCAAGCTCAGCATCCCAGTGGTAACAATCTACGCGACGCTCGGCGACGAGGCCATAGCACACGGCATCAACGAGACGGAGGTGTCCACTGTCATCACCACTCACGACCTGCTGCCCAAGTTCAAGAAGATCCTCGCCAAGACGCCCAAAGTGGACACCATCATATACATGGAGGACCAGCTGCAGACCATCGACCGGGAGGGCTACAAGCCCGGCATCAGGATCGTTGGCTACAAGGAGGTCATACAGAAAGGAATAAACGCCAGCTTTGAGGCGGTGCCGCCGGCGCCGACGGACACGGCCATCATCATGTACACGTCGGGCTCGACGGGCGTGCCCAAGGGCGTCATCCTGTCGCACCGCAACATGCTGGCCACGCTCAAGGCGTTCGCGGACGTCGTGCCCATATACGAGGACGACATGCTCATGGGCTTCCTGCCGCTCGCGCACGTCTTCGAACTATTGGCCGAGAGTCTCTGCATCATTGGTGGTGTCCCCATCGGGTACTCGACGCCGCTGACGATGCTGGACTCCTCCAGCAAGATCATGAAGGGCACCAGCGGAGATGCCACCGTCCTCAAGCCCACCTGCATGACCACAGTGCCGTTGATAATGGACCGCATCAGCAAGGGCATCACGGACAAGGTGTCTCGCAGCGGGCCGTTCGCGAGCGCGTTCTTCCGCTGGGCGTACTCGTACAAGCAGACGTGGATGCGGCGCGGATACGACACGCCCATACTTAACAGGATTATGTTCAGCAAGATCCTGGGCCTGCTGGGCGGGCGGCTGCGGCTGCTGCTGGCGGGCGGCGCGCCGCTGGCGCCGGACACGCACCAGCAGCTGCGCATCTGCCTGTGCTGCGACGTGGTGGCGGGCTACGGGCTCACCGAGACCACGTCGGCCGCCACCGTCATGGACGCGCACGACCGCTCCACCGGCCGCGTGGGCGCGCCCTCGCCCGGCACCGCGCTGCGCCTGCTCGACTGGGCCGAGGGCGGCTACCGCGTCGCCAACAGGCCCTTCCCGCAGGGCGAGATCGTGATCGGAGGTGATTGCGTAGCGGAGGGCTACTACAAGAATCCAGAGAAGACCCGGGAGGAATTCATCGAGGAGGACGGCATTCGCTGGTTCAGGTCTGGGGACATCGGCGAACTGCATCACGACGGCTGCATCAAGATCATCGACCGCAAGAAGGACCTAGTGAAGCTGCAGGCCGGCGAGTACGTGTCCCTGGGCAAGGTGGAGGCGGAGCTGAAGACGTGCCCCATCGTGGAGAACATCTGCGTGTACGGCGACAGCTCCAAGACGTACACCGTGGCGCTGGTGGTGCCCAACCCGCGGCACCTGGCCGAGCTGGCCGCGCGGCTCGGCCTGCCCGACCGGGACTTCGACCAGCTCTGCCACAACACCGCCGTCGAGAAGGCCGTCGTCAAGGAGCTCGCCGACCACGCTAGGAAGTGTGGGCTGGAGAAATTCGAGGTTCCTGCTGCAGTGAAGCTGTGCACGGAGGTGTGGTCCCCCGACATGGGGCTAGTTACCGCCGCCTTCAAGATCAAGCGGAAAGACATCCAGGAGCGGTACAAGGAAGACATCAAACGAATGTACGCCTCCTGA-3’ |
| gme-new-135-5p  5’- CTCATTATTTCGTCTGAAAAGAAA-3’ | Gene 13  5’- ATGAAGAATCTGGAGATGACTCAGATCCGGGAAATAGTGGATTGCATGTATCCAGTAGAATATGCCGCCGGTAGCCTCATCATCAAAGAAGGAGATGTTGGCAGTATTGTATATGTTATGGAAGAGGGAAGAGTGGAAGTGTCTAGAGAGAACAAATACCTCAGCACAATGGCACCGGGCAAGGTGTTTGGTGAACTAGCCATTCTTTACAACTGCAAGAGAACGGCCACAATAAAAGCAGCAACTGATTGTCGGTTGTGGGCCATTGAACGTCAATGCTTCCAGACTATTATGATGAGAACTGGACTCATAAGACAAGCGGAATACACTGATTTCTTGAAGAGTGTGCCGATCTTCAAAGACCTTCCCGAAGACACGCTTATCAAAATTTCTGATGTTTTGGAAGAGACACATTATCAGAACGGTGACTACATTATCAGGCAAGGAGCGCGTGGTGACACGTTCTTCATCATTTCCAAAGGACAGGTAAAAGTGACCCAGAAGCAACCAAACAGTAACGATGAGAAATTCATTAGAACACTAACGAAAGGCGATTTCTTCGGAGAAAAAGCGTTACAAGGAGATGACCTTCGAACAGCCAACATCATCTGTGACTCACCAGAAGGTTGTACATGCCTTGTAATTGATCGGGAGACCTTCAACCAACTCATTTCGACCCTAGATGAGATACGTACCAAATATAAAGACGAAGGCGATAGTAGACAGAGATTAAATGAAGAATTTGCCAATTTGCGTTTATCAGATCTTCGTATCATAGCCACCCTCGGTATCGGCGGTTTCGGAAGAGTGGAACTTGTGCAAATACAAGGAGATCCGAGTCGATCGTTCGCCTTGAAGCAGATGAAGAAAGCCCAAATCGTTGAAACGAGACAGCAGCAACATATTATGTCAGAAAAGGAGATAATGTCAGAAATGAACTGCGAATTCATAGTGAAGCTATTTAAGACATTTAAAGATCGCAAATACTTGTATATGTTGATGGAGACATGCCTCGGAGGAGAGTTGTGGACTATTTTAAGAGACAGAGGCCAGTTTGATGATGCCACAACAAGGTTCTATACCGCTTGCGTTGTAGAAGCCTTCCATTATCTACATTCTAGGAATATCATTTACAGGGATCTCAAACCGGAAAACTTATTATTAGACTCCAAAGGTTATGTGAAATTAGTCGATTTCGGTTTCTCCAAGAAGCTGCAAGCGAGCCGTAAGACTTGGACATTCTGTGGTACTCCTGAGTATGTTGCACCCGAAGTCATTATGAATAGAGGTCATGATATCAGCGCAGACTATTGGTCATTAGGTGTGCTAATGTTCGAGCTGCTGACAGGATCACCTCCATTCACCGGAGCTGACCCAATGAAAATTTATAACAAGATTCTCAAGGGTATTGATGCCGTGGAATTCCCCCGATCGATCACCAGAAACGCAGCTAATCTCATAAAGAAATTGTGTCGTGACAACCCTGCCGAACGGCTTGGGTATCAGAGAGGAGGCATCACTGAGATACAGAAACACAAATGGTTTGACGGCTTCAACTGGGAGGGCCTGGCTCAGCGCACCTTAGAGCCGCCGATCACACCAGTCGTAAAGTCCGCTGTCGATACGCACAACTTCGACCAGTATCCGCCTGATGCAGATGAACCGCCA-  3’ |
| gme-new-161-3p  5’- TACTTGTCTAGAGGGCGTGA-3’ | Gene 14  5’- ATGAAATTTACAATTGTCATAAATATTACAAATAAGGATGCAGCAACTACATCTGAGTTATTTAATAAAAAACCAGATAAGTCCGTAACCATTTTTGATTTGGTTATACACTTTTATAATTTTGACTATCAGAATAGTACTACACATGTACCTGAACACTCATACATAGCAATGACATCTGGCAGCACTGGAGAACCAAAGCACATACAAGTGCCTGTGCAATGTATTCAACCAAATATAGATGATTTAACTAAATTATTTAATATTACTGCTGATGATATAATATATTTCTCTACACCACTAACATTTGACCCATCCATGATAGAGATACTGCTCGCCTGTATGAATGGAGCCTCTCTACTTATTGCACCTGAAAAAGCAGACATATTATTCCCCAACAACAAAGAGAATTCTGTGACATTTTGGCAACTTACACCATCACGATTTTTTCAGCATTCAAATTCTGATATCAAGAATAAAATATTAAGTGCAAATTCAACATTGAAAATACTAGCTTTAGGTGGTGAGCCATTAAATGGTGTGAAGAGACTAAAGGAATTGAAAGATTGGGATAATAAAACTAGAATATTCACATTGTATGGAGTAACTGAAATGTCATGCTGGGCTTGTGTTGCTGAGTTAGATCTTAACAAAATACTAACTGACAAAGAAATACCATTAGGTAACTGTCTGTCAGAAACAGAATTACATGTGGAATCAAATGATGACAATAAAGAATGTGGAAAAATTATTTTAGTAAGCAAAACAAGAAAGTGTGTTATATTAAACAAAACCATTGGAAATGAAGATGAAAATTCTTTAAAATTTATTGACACTGGAGATTTAGGTGAAGTAAGAAATGGCACTGTGTATTATCGGGGCCGCAAAGATGATATTATTAAAAGATTTGGACACAAAATTAATTTACAGTTTATTGAATCAACTATAATGCAATGTCCGAGTGTGAAAACAAGCTCCTGTATCTGGCTCCCAAAATCATTACTTCTGATTGCCTATTTCTCATCAGAAACACTTAGCAGCCAAGAGTTGTCCAACTTTTTGAAATGTAAACTTGATGATAAGCACTGGCCAGATAAAATAATTAGAGTTGACAATTTACCAACAAATCCTCATGGGAAGATATCTAAATTGATATTATCTAAAATGTATGAAAAAACAATGAACACACCACAGACATTAGATTCCTTAAAAGTGAGTTTCTTAAAGGAACTTCAAGCTGTAATGGGTCAACACTTCACTTATGATCAAATTAAAAGTAAAGACTTCTTTGCCATTGGTGGCACATCTTTTCTAGCGATATCTATGTGTAATAAGCTTTCACTACTTTGTCCAAAATTTGGTAAACTAATTCTCCCTTACTTGATGTCTCAAAAAAATACTATAGATGATATTATGCAATTGGCATATAAAGAAATACATGTTGATGAAATAAAAGTTAAGAAAAAAATTAAAAGGTCACGGTCAGATGCAGGTGGTTATGTAGAGAGTCAGTCTTACAAAAGAACTAACACAAAAAGTCTGACAAATCCTGTAAAATTCATTGTGTTATGGTCATATGATACTGGAAAATGTGTAGATGCTTCTCCATCTTTATTTCAAATAGGATTCAATTTATATGTGACAGTCGGGAGTCATTCAGGGAAGATTATAGTCATGGATGCGATATCTGGAATATTGCAGGGAATGGTAACAGTAAAATCACGTGTTGAAGCATCTGTATTTTGTTACCACAAGAGAGACATGTCGCCGTGCGGTGTGGTTGGCACTTACGATGGCACAGTGGTATGCTTCCAATTAGAAACATGCAAAGAGTTGTGGAGAATCAACATTGGATCAATGATAAAAAGTAAAGCAACATGTTGCAATGATCTACTCTACATTGCTTCCTATGATGGAAAGATAAGATGTATAGATATTGCGATAGGGGTAATCAAAGAGACTATATATGTGGCAGATCAAGGTATATCAGCTGATCTAGTACTTGCTAAAAACAAGTATGTGTTAACCAGTACGCTGTCTGGTGTGTGTGCAAGTATACATACTCTAACCAATACTGTGGCTTGGCGCTGCACACTGAGTAGTCCAGTATTTGCAAGTCCTGTGCTTTACGATGACGACAAGTATGTGGTATTCGCGGAAGTTAATGGTGAAATACATTGCAGGACCGTTGAAAAGGGTATTAAGATATGGAATTATCAAGGAGCAAGAGGTAACATTTTTTCCTCCCTCTACATAAAAGAAGTTGATAAACTGAAATGGCAAATGGTTTTTGGCTGTCACGATAACAAAGTTTACAGCATTAATATCAAGAATTTCCAACCTAGCTTGAATTGGAAAGCACAACTCACATCACCTGTATACTCCACTCCATGTGGTCTAAGTGACAAATTAATACTTGCTGCCTCTAATAATGGCAGGTTATGCGTTATAGATGAAGAAAACGGGATAATATTGGCAGAACATCATCTGCCAAATGAAACATTTTCATCACCAGCAGTTTATGGAGATTACATATTCATTGGTTGCAGGAATGACCACCTTTACTCTTTGAAATATATTTTAAATTTATAA-3’ |
| gme-new-122-3p  5’- TAATGCCTTCCTCTAACTACA-3’ | Gene 15  5’- ATGGCGTCTGATACCTTAGTACCTATAGAATCAAATCCTGAGGTTATGAATAAATTCCTTCAAAAATTAGGTGTTCCATCTAATTGGAGCATAGTCGATGTAATGGGCTTAGATTCTGAGATGCTGTCTTGGGTTCCTCGTCCGACTATTTCTGTTATGCTGCTGTTTCCTGTATCTGCTGCATATGAAGATCATAAAAAGAAAGAGGAAAGTGAAATATTGGCTAAGGGCCAAGAAGTTTCAAGTGACATTTTTTATATGAAACAAAATGTAAGTAATGCTTGTGGCACTGTAGCTCTGGTACACAGTGTCGCCAACAATTATGATAAAATCCAGCTTTCTGATGGCCCTATGAAAAAATTTATAGAAGAAGCCAAACCATTAGATGCTGCTGCTCGAGGAACTCTGTTTGCAAAGACTGAAGGCATTATCAATGCTCATAAAGAATTGGCTCAAGAGGGTCAAACTAATACCCCCAGTGCCGAAGAACCTGTTGATCATCATTTTGTAGCATTTGTACACAAAAATGGAGCATTATATGAATTAGATGGCAGAAAGGCTTTCCCTATCAATCATGGACCTACTACACCAGATAATCTATTAGAAGATGCTGCTAAAATTTGCAAAGAATTCATGGCTCGTGATCCTAATGAAGTTCGTTTTACTGTGATGGCTTTAGCAGCTTCCAACTAA-3’ |
| gme-new-82-5p  5’- TCTGTTCGTATTGTCAAGTATA-3’ | Gene 16  5’- ATGAGAGATCCGCGCACGTTCGCAGTCATGCATAACGTTAGGCCACTGCAATTTGTTACGGAGTCGGCCGGTGGGCACCGGAATCGTTCGATGTCATTGTCTACACACCACCACAATGGAGCGCCGAGACCGGAGCCTAGATATTCTTTGCAACTTCAGACTGATGAAAACAGCTACCTACTTCGTTTGAAGATAATAGGTGCTACGTCATTGGCCAAAAAAGATATATTCGGTGCTAGCGACCCCTATGTGCGTGTGGAATTGCAGAAACTCGACAGCGACGTCACCCTCGAGACGTTTCTTACAAAAACCAAGAAAAAGACATTAAATCCAGTATGGAATCAGGAGTTTGTATTTAGGGTGAACCCCCGCGAGCACAAGCTGCTGATCCAGGTGTTCGACGAGAACCGGCTGACGCGTGACGACTTCCTCGGCATGGTGGAGCTGGCGCTGGGCGCCGTGCCCACCGAGAGCGCGGCCGCCGCCCGCCCGCCGCCCCTCAAGTACCCGCTGCGCCCGCGCAGCGCGCGGTCTCGGGTCCGCGGCCATATCGAGGTGTACGCGGCGCTGGTGGGGCGGGTGGGCGAGCCGGGCGTGGCGGCGGCCGCCGAGCGCGGCGACGACTGGGAGCTGGTGCACGCCGCGCCCGCCGCCGGCGAGGTTCACTCGACGGTGGTGGGTGATCCGTTACCGCCCGGCTGGGAGGAGCGACAGGACGGCAACGGGCGGACTTACTACGTGAACCACATCGAGCGGTCCACGCAGTGGGAGCGGCCTACATTCACCCGCAACCAAAGTGTGGAGTCGCAAGCTGAACGTATGGAGACGGCGGTCACCGAGTTCCAGCGGCGGTTCCACATCTCTGCGGACGAGGAGCACTCTAGTCCTGCGTCCTCGCAGCACCAGGACGAGACCGACGGGTCGACCGAGAGCACGCGCAGCAGCAGCGCGAGTAGTACGCAGAGTCAGAATCTACCGAACGCTGATGGATTACCACCGGGATGGACGATGCAGAAAGCGCCCAACGGCAGGATATTCTTCATCGACCACAACCAGAAAACAACGACGTGGATAGACCCCAGAACAGGGTGCGCATCAAGCTTGCCGAGTGCGGCGGCGGCGACGGCCGGTGCTGAAGCGGACGAATTGGGAGCGCTGCCAGAAGGCTGGGAGGAGAGGGTCCACACCGACGGCAGGATCTTCTTCATCGACCACAACACGCGTACAACCCAGTGGGAGGATCCGCGGCTATCAAACCCTCAAATCGCGGGTCCCGCCGTGCCGTACTCGCGAGACTATAAGCGCAAATATGAGTACCTGAAGAGCCAGCTACGCAAGCCGAGCAACGTGCCCAACAAGTTCGAGATCAAAGTGCGTCGTAACTCGATACTGGAGGACTCATACCGCATAATCACCTCCGTGAACCGCATCGAACTGCTGAAGACCAAGCTGTGGGTGGAGTTCGAGTCGGAAGTGGGACTAGATTACGGAGGTCTGGCGCGCGAGTGGTTCTTCCTTCTGTCCAAGGAGATGTTCAATCCTTACTACGGGTTGTTCGAGTACTCGGCGATGGACAACTACACGCTGCAGATCAACCCGAACAGCGGCGTCTGTAACGAGGAACACCTGAGCTACTTCAAGTTCATCGGTCGCGTAGCCGGCATGGCGGTCTACCATGGGAAATTGCTCGACGCATTCTTCATCCGTCCATTCTACAAAATGATGCTGAGCAAGCCCATAGAGCTGCAGGACATGGAGTCGGTCGACTTGGAGTACTATAACTCGCTCATATACATCAAGGAAAATGATCCGTCGGAATTGTACCTAACGTTCTCGGTGGACGAGGAACAATTCGGCAAGACCATACAGAGGGACCTCAAACCAGGAGGTGCTAACATACCAGTCGATGAAGAGAACAAGGATGAGTATATCAAGCTGGTGATCCAATGGCGGTTCGTCAGTAGAGTCCAAGAGCAGATGTCATCGTTCCTCGAAGGGTTCGGGGCGCTGGTGCCGCTGAACCTGCTGAAGATCTTCGACGAGCACGAACTGGAGCTGTTGCTGTGTGGAATCCAGCATATAGACGTGCGAGATTGGCGCGCCAACACACTGTACAAGGGAGACTATCATGCCAATCATATAGTGGTCCAATGGTTCTGGAGGGTAGTGCTGTCGTTTTCGAACGAGATGCGGTCGCGGCTGCTACAGTTCGTGACGGGTACGTCGCGGGTGCCCATGAACGGCTTCAAGGAGCTGTACGGCTCCAACGGGCCGCAGCTGTTCACCATCGAGCGCTGGGGCAGCCCCGACAACTACCCCAGGGCGCACACCTGTTTCAATCGAATCGACCTACCTCCATACGAGAGTTATCAGCAGCTTCGCGAGAAGCTAGTCAAAGCGATCGAGGGCTCACAAGGCTTCGCCGGCGTCGACTGA-3’ |
| gme-new-117-5p  5’- TTTTTCACCAGCGAAGTCAGA-3’ | Gene 17  5’- ATGCAGATATTTGTCAAAACATTAACTGGGAAAACCATCACATTGGAAGTAGAACCATCGGATACTATTGAAAATGTGAAAGCCAAAATTCAAGACAAGGAAGGCATTCCCCCAGACCAGCAACGACTCATTTTTGCAGGCAAACAATTGGAGGATGGCCGTACACTTTCAGATTACAACATCCAGAAGGAATCTACGTTGCACCTTGTTCTTCGTCTAAGAGGTGGTATGCAGATCTTTGTAAAGACATTAACAGGAAAAACTATCACCTTGGAGGTTGAACCTTCTGATACTATTGAAAATGTAAAGGCTAAGATTCAGGATAAAGAGGGCATTCCACCAGACCAACAACGTCTTATCTTTGCTGGCAAGCAGCTAGAAGATGGACGCACACTCTCTGATTATAACATCCAAAAAGAATCAACATTACATTTAGTATTACGACTTCGTGGTGGTATGCAAATTTTCGTAAAAACCTTGACTGGTAAAACAATCACATTGGAAGTTGAACCCTCTGATACTATTGAGAATGTGAAAGCCAAAATCCAAGATAAAGAGGGTATTCCACCTGACCAACAACGTCTTATCTTTGCTGGCAAGCAGTTAGAAGATGGACGCACACTCTCTGATTATAACATTCAAAAAGAATCTACTTTACATTTAGTATTACGACTTCGTGGTGGTATGCAAATTTTCGTAAAAACCTTGACTGGTAAAACAATCACATTGGAAGTTGAGCCATCTGACACTATTGAGAATGTGAAGGCTAAAATTCAGGATAAAGAGGGCATTCCACCTGACCAACAACGTCTTATCTTTGCTGGCAAGCAGTTAGAAGATGGACGCACACTCTCTGATTATAACATTCAAAAAGAATCTACTTTACATTTAGTATTACGACTTCGTGGTGGTATGCAAATTTTCGTAAAAACCTTGACTGGTAAAACAATCACATTGGAAGTTGAACCCTCTGATACTATTGAGAATGTGAAAGCTAAAATCCAAGATAAAGAGGGTATTCCACCAGACCAGCAGCGACTTATCTTTGCTGGCAAGCAACTTGAAGATGGGCGTACACTTTCTGATTACAATATACAGAAGGAATCAACACTACATCTTGTATTACGTCTTCGTGGTGGTATGCAAATTTTCGTAAAAACCTTGACTGGTAAAACAATCACATTAGAAGTTGAACCCTCTGATACTATTGAGAATGTGAAAGCCAAAATCCAAGATAAAGAGGGAATTCCTCCAGACCAGCAGCGACTTATCTTTGCTGGCAAGCAACTTGAAGATGGCCGTACACTTTCCGATTACAACATTCAAAAAGAATCTACTTTGCATCTTGTACTAAGGTTGAGA-3’ |
| gme-new-160-5p  5’- GTCATTCAGCCTGCCAGCATTGCT-3’ | Gene 18  5’- ATGACGGATTCTACAGTTGTGTTTGTCAATTCTGGACAGCCGCCCCCTCCATATATTGCTCAGCCCGTGGCGCCCCCAGTCGCAGTGGTAATGACTGGGCCAGTGGGCTCAGAGCCTATAATCATGGCCTGCCCCTCGTGCCGTCACCAGATCGCAACGAGAGTTGAAAGAGCAGCATCATCAAAAACTCATATCATAGCTTGCTTATTGTGTTTATTTGTATGCTGGCCATGCGTTTGTGTACCATACTGTGTGGATTCGTGCAACAATGCCAATCATTACTGCCCTAACTGTAACGCGTATATAGGCAGTTATAATTTTTAA-3’ |
| gme-new-160-5p  5’- GTCATTCAGCCTGCCAGCATTGCT-3’ | Gene 19  5’- ATGACGGATTCTACAGTTGTGTTTGTCAATTCTGGACAGCCGCCCCCTCCATATATTGCTCAGCCCGTAGCGCCCCCAGTTGCAGTGGTAATGACTGGGCCAGTGGGCTCAGAGCCTATAATCATGGCCTGTCCTTCATGCCGTCACCAGATCGCAACGAGAGTTGAAAGAGCAGCATCATCAAAAACTCATATCATAGCTTGCTTATTGTGTTTATTTGTATGCTGGCCATGCGTTTGTGTACCATACTGTGTGGATTCGTGCAACAATGCCAATCATTACTGCCCTAACTGTAACGCGTATATAGGCAGTTATAATTTTTAA-3’ |
| gme-new-136-3p  5’- TCAATTTGGTTTTAATCTGAAT-3’ | Gene 20  5’- ATGGGAAGAAAATCACAGTCAAAACTAAGTTCTAAAAAGAATGCTAACAGGGAAAATAATAGAATAGTACAAAAGCGCAAAGAACTTGCGATTCTTGCGGATAAACTACTTCGATTAACAAGTATAATAACTCAAGTGTCCAACATTGGAAATAGCTGGGAACTTCACAAACAAATTGAAGCAGTCATCAAAGAAATATTAATTATTGAAGCACCATTCAATATTAAAACAAAACAAAATCCACGTCACTTAAACATAGAAAATTTCTTAAAATGGTTAAACGAAAATGTAGCTACATTTGAAGGTGTGGAAATTGGTGAATTTGAAGGCTATGAATTTGGTTTAAAAGCAACAAAGAACTTTAAAGAGGGTTCATTATTGCTCACAGTGCCCAGTAAACTTATGTTGACTGTACAAAATGCCAAGGAATCTGAACTATCGGATTTTATCAGTATGGATCCTCTTCTACAAAATATGCCCAACATAACATTGTCATTATTTTTATTATTGGAAAAAAATAATCCAGATTCTTTTTGGAAGCCATATATTGATATTCTGCCAGAAAAATATTCAACCATCCTTTACTTCACCGCTGAAGAACTAGCTGAACTCAGGCCTTCTCCAGCTTTTGAGTCAGCTCTAAAACTTTATAAAAGTATTGCAAGACAATATGCATATTTCTACAATAAAATACATACTTCGAATATACCAGTCTTGAAAAATCTCCAAGAAATTTTCACATTTGATAACTACAGGTGGGCTGTGTCCACTGTAATGACGCGACAGAACATGATACAGCTGGATGACTGTGACGTCACGTCATTTATACCCTTATGGGATATGTGTAACCATGAACACGGAAAGATCACAACCGACTATAACAAGGAACTAAATAGAGGCGAATGCTACGCACTGCGCGACTTCCAACAAGGCGAACAGATCTTCATATTCTACGGTGCGAGATCCAACGTTGATCTGTTCTTGCACAATGGTTTTGTATATCCAAAGAACCAATACGATAGTCTATCCCTATCTCTGGGTATAAGTTCAAGTGATCCACTGCGCGAAACGAAGCTATCCTTATTGAGCAAACTGGGTCTCGCCGGAGTGACACATTACAACCTATACCGTGGTGAAACACCCATCAGCGCTGAGCTGCTCGCCTTCATCAGGATATTTAATATGAATCAGGAGGAATTGGCAAAGTGGTCGAGCCAGGGTTTGCCCAGTGACCTGGTATCGTCCGAGTCCTCTAGCGTCGATGCAGTGGGCGCGGACGTCGACCGGCGCGCGTACAAGTACCTGCTAACCCGTTGCGAACTCATACGTGCTTCATACAACAAAACTAATAGCGATACAGAACAAAACGGCCCTGAATCTACACACAGGAAAAATATCAAACTGTTGAAGGAATGTGAAGTACAAATATTGGAAGGTGCCATAAAATATCTTGAGAACGTTTTACAAAAACTACCCACCGTGAAATAA-3’ |
| gme-new-106-5p  5’- CCTTGTCATTCTTCTTGCCCAGT-3’ | Gene 21  5’- ATGAAACTTAATATTAACACACAAATTAATGGATTAAATATTACGTTAGTGCCTTACAGAAAACATCATGTACCAAAATATCATATGTGGATGAAATCAGAGGAACTACAAAGACTCACTGCATCAGAACCTCTGTCCTTAGAACAAGAGTATGAAATGCAAAAATCTTGGCAAGACGATGATGACAAATGTACGTTTATAATATTGCAGAAGAATACAGAAACAAATGAAATAGATTCAATGATAGGTGATACAAATATATTTGTAACAGACAAAGAAAATTATATTGGAGAAATAGAAATAATGATAGCAGAAGAGTCTGCAAGAGGAAAGAAACTTGGTTGGGAAGCTGTAATTTTAATGTTGATTTATGGTATTAAATATATCAATCTTAAAATGTATGAAGCAAAAATATCATTAAGTAATATTATAAGTATACAAATGTTTAAAAAGTTAGGTTTTAGTGAGAAATCAAAAAGT-3’ |

| Supplementary Table S4. Stem-loop structures of 37 novel G. mellonella miRNAs indicating mature miRNA sequence (red color), miRNA star strand sequences (violet color), loop (orange color), and star sequence (blue color) | |
| --- | --- |
| **Novel miRNA** | **Structure** |
| gme-new-90 | 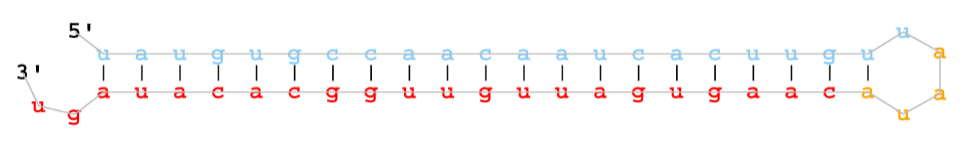 |
| gme-new-80 | 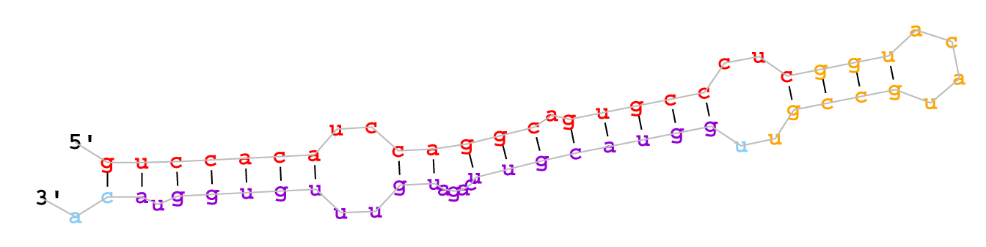 |
| gme-new-72 | 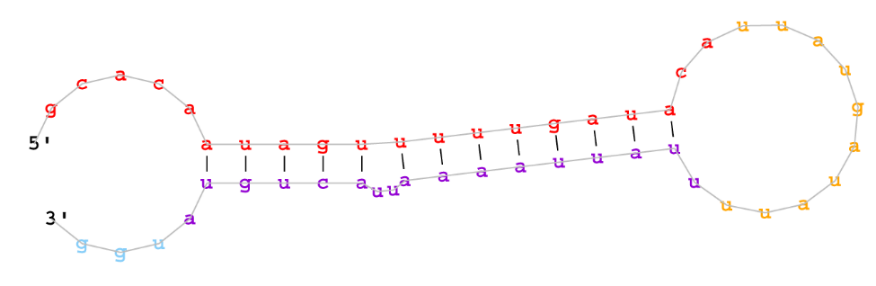 |
| gme-new-78 | 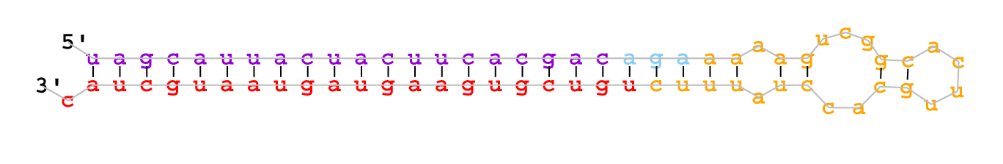 |
| gme-new-161 | 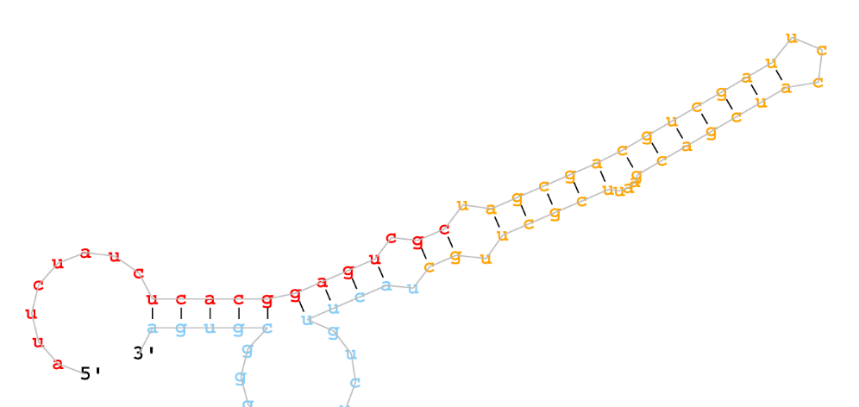 |
| gme-new-137 | 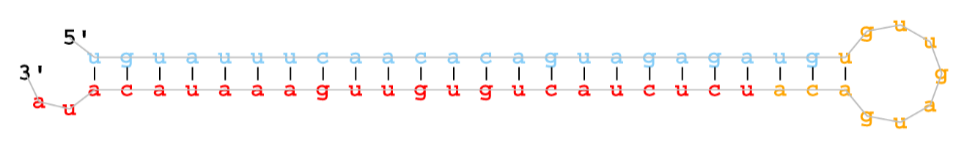 |
| gme-new-136 | 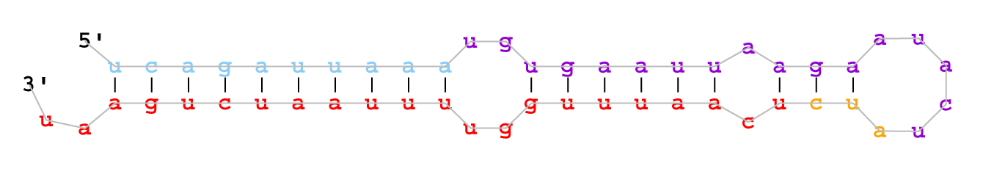 |
| gme-new-135 | 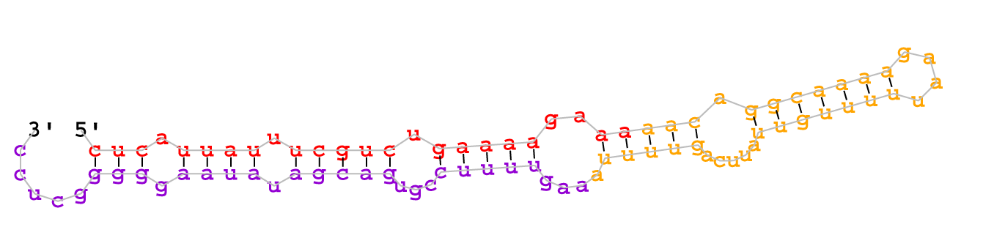 |
| gme-new-89 | 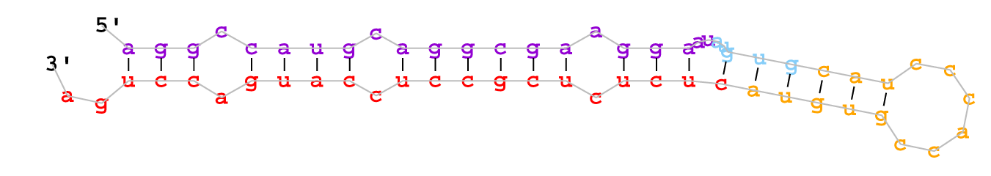 |
| gme-new-88 | 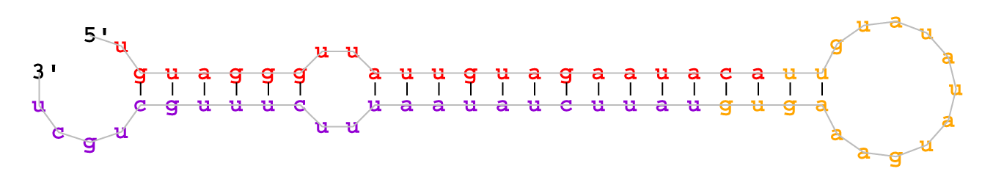 |
| gme-new-70 | 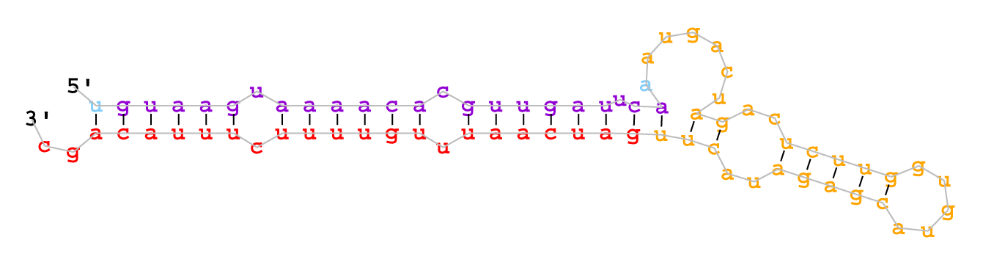 |
| gme-new-157 | 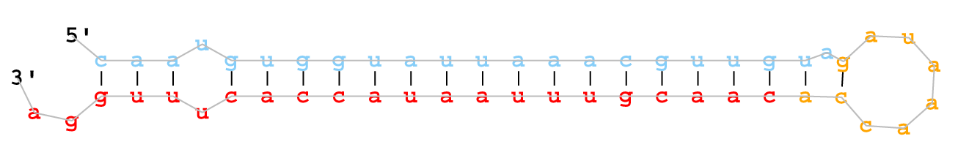 |
| gme-new-156 | 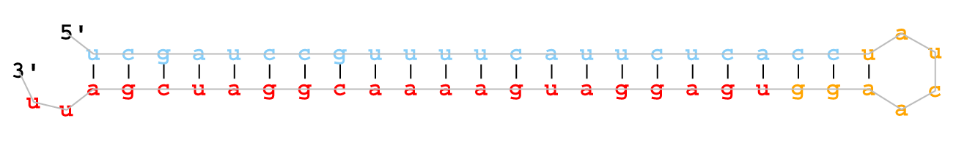 |
| gme-new-150 | 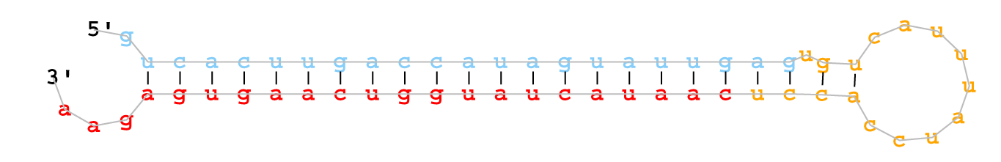 |
| gme-new-147 | 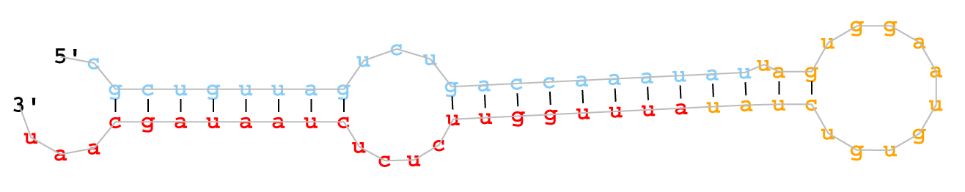 |
| gme-new-140 | 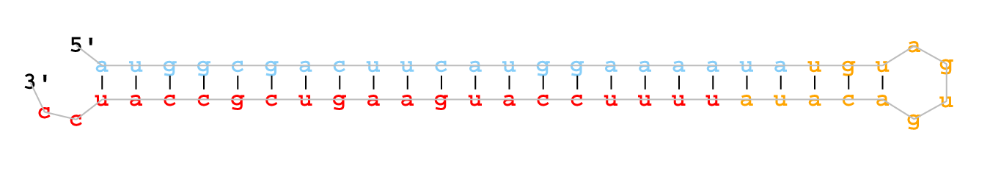 |
| gme-new-122 | 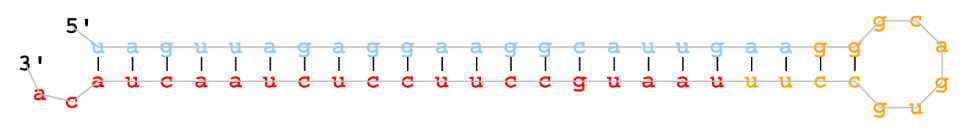 |
| gme-new-117 | 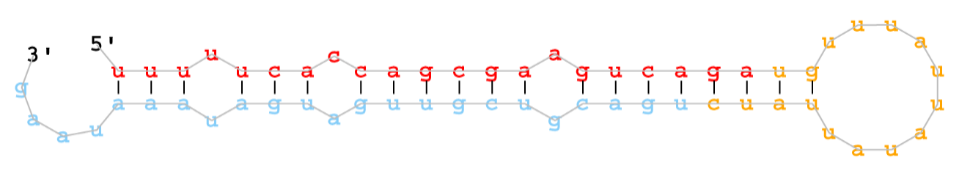 |
| gme-new-160 | 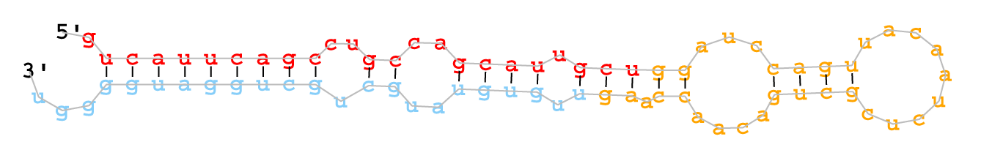 |
| gme-new-123 | 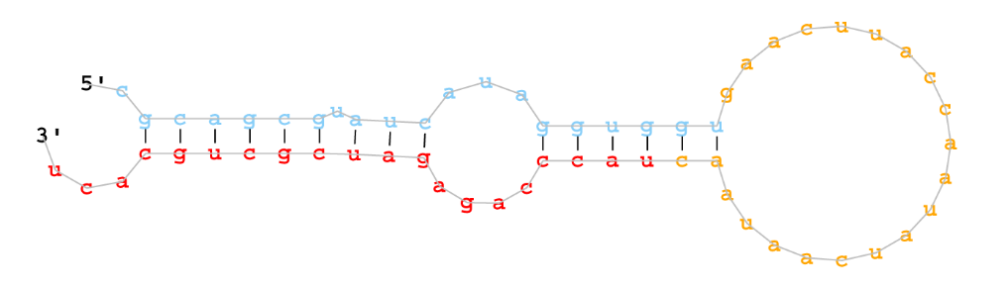 |
| gme-new-97 | 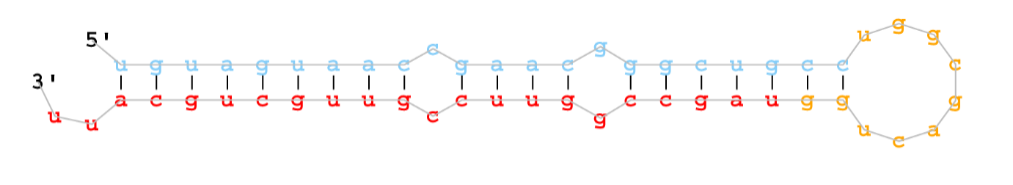 |
| gme-new-93 | 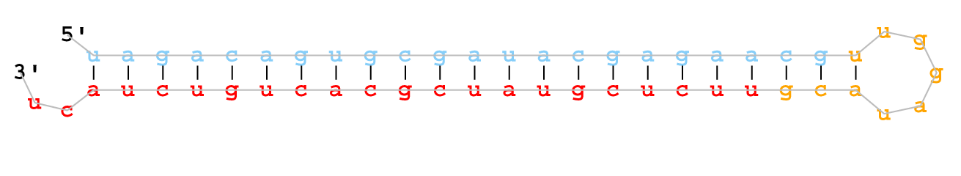 |
| gme-new-89 | 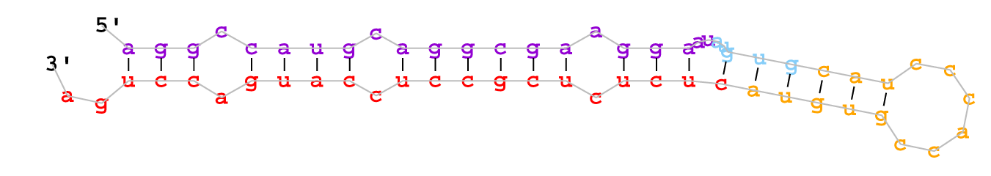 |
| gme-new-82 | 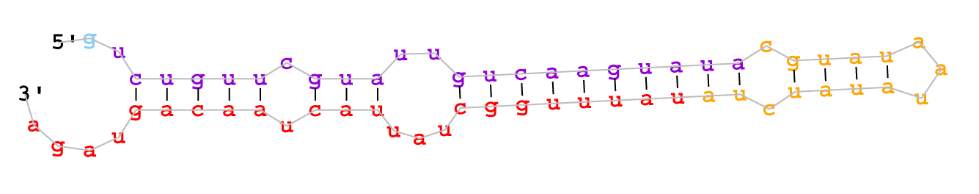 |
| gme-new-61 | 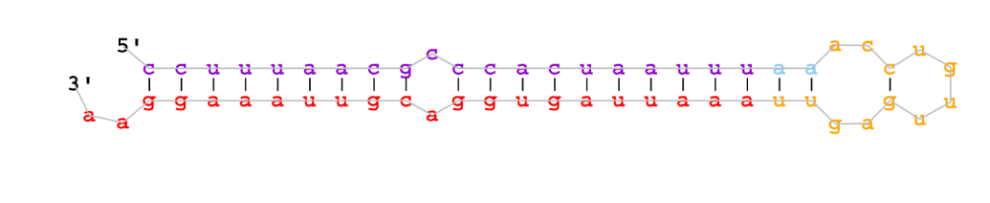 |
| gme-new-149 | 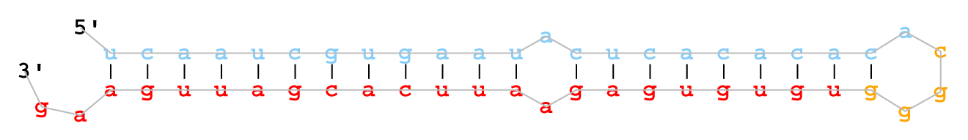 |
| gme-new-146 | 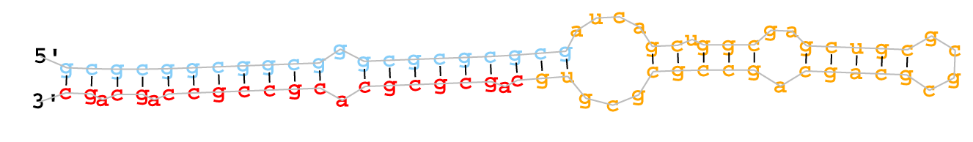 |
| gme-new-144 | 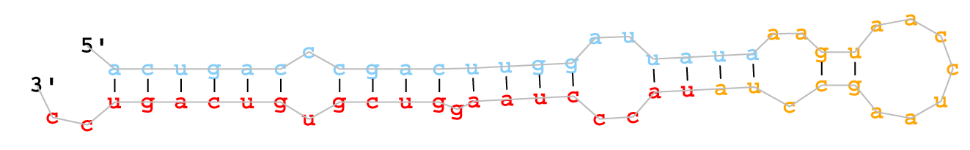 |
| gme-new-136 | 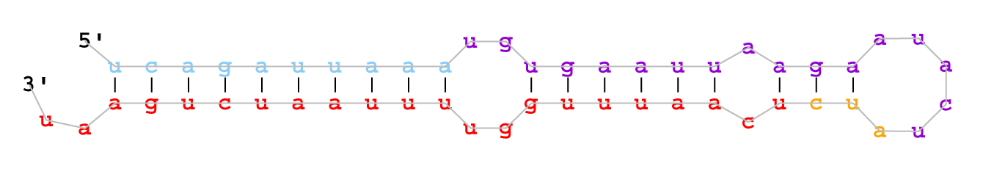 |
| gme-new-107 | 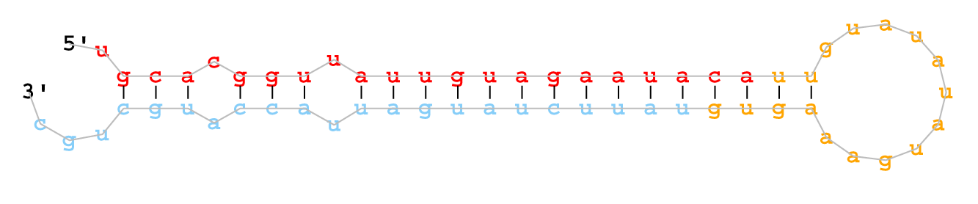 |
| gme-new-90 | 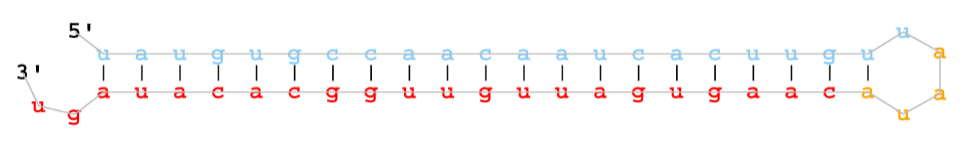 |
| gme-new-81 | 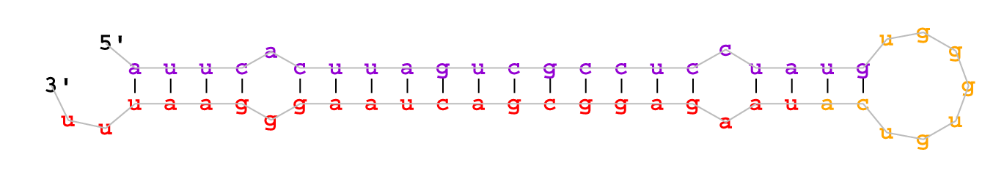 |
| gme-new-52 | 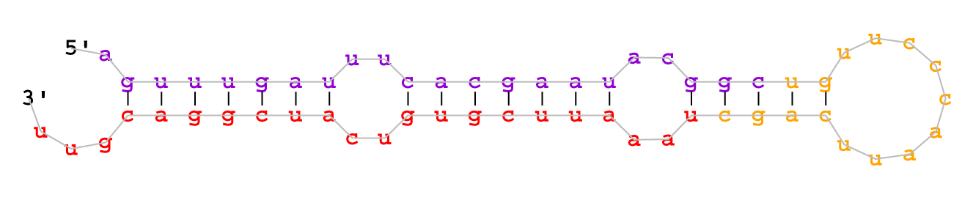 |
| gme-new-153 | 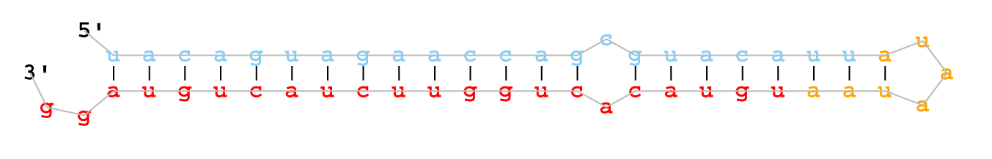 |
| gme-new-141 | 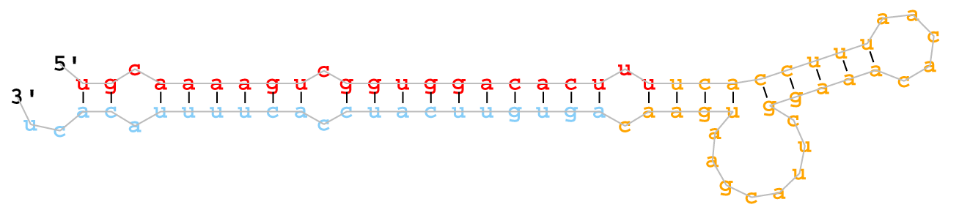 |
| gme-new-137 | 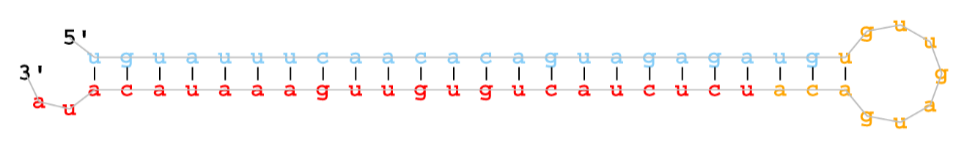 |
| gme-new-135 | 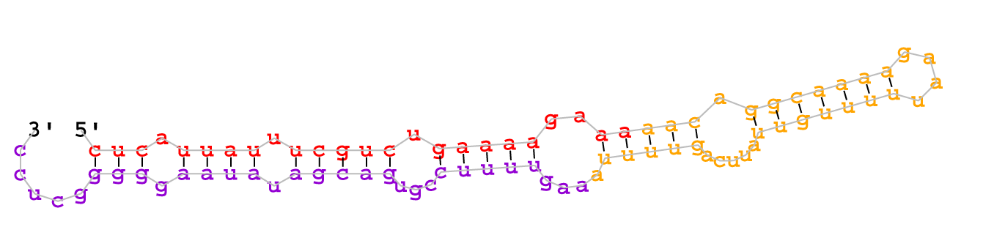 |
